# Two DNA-binding One Zinc Finger transcription factors, MdCDOF3 and MdDOF3.6, accelerate leaf senescence by activating *cytokinin oxidase MdCKX7* in response to sorbitol signaling in apple

**DOI:** 10.1093/hr/uhaf120

**Published:** 2025-04-29

**Authors:** Wang-Jiang Zhang, Chang-Ning Ma, Lian-Da Du, Ying Xiang, Fan Xiao, Ya-Ting Liu, Chu-Kun Wang, Wan-Kun Li, Ting-Ting Zhao, Da-Gang Hu

**Affiliations:** National Research Center for Apple Engineering and Technology, Shandong Collaborative Innovation Center of Fruit & Vegetable Quality and Efficient Production, College of Horticulture Science and Engineering, Shandong Agricultural University, No. 61, Daizong street, Tai’an, Shandong 271018, China; National Research Center for Apple Engineering and Technology, Shandong Collaborative Innovation Center of Fruit & Vegetable Quality and Efficient Production, College of Horticulture Science and Engineering, Shandong Agricultural University, No. 61, Daizong street, Tai’an, Shandong 271018, China; National Research Center for Apple Engineering and Technology, Shandong Collaborative Innovation Center of Fruit & Vegetable Quality and Efficient Production, College of Horticulture Science and Engineering, Shandong Agricultural University, No. 61, Daizong street, Tai’an, Shandong 271018, China; National Research Center for Apple Engineering and Technology, Shandong Collaborative Innovation Center of Fruit & Vegetable Quality and Efficient Production, College of Horticulture Science and Engineering, Shandong Agricultural University, No. 61, Daizong street, Tai’an, Shandong 271018, China; National Research Center for Apple Engineering and Technology, Shandong Collaborative Innovation Center of Fruit & Vegetable Quality and Efficient Production, College of Horticulture Science and Engineering, Shandong Agricultural University, No. 61, Daizong street, Tai’an, Shandong 271018, China; National Research Center for Apple Engineering and Technology, Shandong Collaborative Innovation Center of Fruit & Vegetable Quality and Efficient Production, College of Horticulture Science and Engineering, Shandong Agricultural University, No. 61, Daizong street, Tai’an, Shandong 271018, China; National Research Center for Apple Engineering and Technology, Shandong Collaborative Innovation Center of Fruit & Vegetable Quality and Efficient Production, College of Horticulture Science and Engineering, Shandong Agricultural University, No. 61, Daizong street, Tai’an, Shandong 271018, China; National Research Center for Apple Engineering and Technology, Shandong Collaborative Innovation Center of Fruit & Vegetable Quality and Efficient Production, College of Horticulture Science and Engineering, Shandong Agricultural University, No. 61, Daizong street, Tai’an, Shandong 271018, China; National Research Center for Apple Engineering and Technology, Shandong Collaborative Innovation Center of Fruit & Vegetable Quality and Efficient Production, College of Horticulture Science and Engineering, Shandong Agricultural University, No. 61, Daizong street, Tai’an, Shandong 271018, China; National Research Center for Apple Engineering and Technology, Shandong Collaborative Innovation Center of Fruit & Vegetable Quality and Efficient Production, College of Horticulture Science and Engineering, Shandong Agricultural University, No. 61, Daizong street, Tai’an, Shandong 271018, China

## Abstract

Leaf senescence, an essential component of the plant life cycle, seriously affects the productivity of numerous commercial crops, with cytokinins serving as crucial regulators in delaying this process. Here, we observed that apple (*Malus domestica*) leaves exhibiting deficiencies in sorbitol synthesis due to antisense inhibition of *ALOSE-6PHOSPHATE REDUCTASE* (*A6PR*) presented an increase in cytokinin content and exhibited a delay in leaf senescence, in contrast to wild-type (WT) leaves. Transcriptome analysis indicated that the expression of *cytokinin oxidase 7* (*MdCKX7*), encoding a key enzyme in the cytokinin degradation pathway, was significantly downregulated in the *A6PR* antisense lines. Functional verification confirmed that *MdCKX7* facilitated the degradation of cytokinin and accelerated leaf senescence. Moreover, this leaf senescence phenotype was exacerbated by the co-expression of two DNA-binding One Zinc Finger (DOF) transcription factors, *cycling DOF factor 3* (*MdCDOF3*) and *MdDOF3.6*, along with *MdCKX7*. Further biochemical and phenotypic analyses demonstrated that MdCDOF3 and MdDOF3.6 bind directly to the promoter region of *MdCKX7*, thereby transcriptionally activating its expression. Intriguingly, the expression of *MdCDOF3*, *MdDOF3.6*, and *MdCKX7* is cooperatively induced by sorbitol. These findings demonstrate that the MdCDOF3/MdDOF3.6-MdCKX7 regulatory module orchestrates leaf senescence by facilitating cytokinin degradation in response to sorbitol signaling, revealing a mechanism by which sorbitol signaling modulates leaf senescence specifically through *MdCKX7*-mediated cytokinin degradation in apple plants.

## Introduction

Leaf senescence is the final developmental stage of plant leaf life directly affecting many important agronomic traits [1]. And leaf senescence is caused by a series of factors, such as phytohormones, natural death, and external conditions, such as darkness, temperature, nutrients, drought, etc [2]. An important sign of leaf senescence is the yellowing of leaves, which is mainly caused by chlorophyll degradation [3] and also accompanied by a weakening of the antioxidant capacity and an increase in oxidative damage. Apple (*Malus domestica*) is a deciduous fruit tree whose yield and fruit quality are greatly affected by leaf senescence, and premature leaf senescence can lead to a reduction in plant nutrient supply and hence yield and fruit quality [4, 5]. Therefore, slowing down leaf senescence is essential for crop production.

Cytokinins are important phytohormones, which promote plant cell division, and the cytokinins identified so far include 6-(γ,γ-dimethylallylamine)-purine, 6-benzyladenine, and zeatin [6]. The first step in cytokinin synthesis is a rate-limiting step, catalyzed by isoprenyltransferase (IPT) [7]; It can be produced throughout the plant and exists as adenosine phosphate-IPTs (ATP/ADP IPTs) and tRNA-IPTs [8]. Adenosine phosphate-IPTs (ATP/ADP IPTs) are responsible for the synthesis of isopentenyl adenine (iP)-type and trans-zeatin (tZ)-type cytokinins, and tRNA IPTs are mainly used for cis-zeatin (cZ)-type cytokinin synthesis [9]. And active cytokinins can be cleaved irreversibly by the cytokinin oxidase (CKX) [10]. Cytokinins play significant roles in many crucial biological processes including development and cell division. During leaf development, cytokinins promote chlorophyll synthesis and prolong the photosynthetic period of leaves [6]. Therefore, cytokinins are considered as negative regulators of leaf senescence in plants [11].

The roles of transcription factors in cytokinin synthesis and degradation have been widely reported, and rice OsDOF11 binds directly to the promoter region of *OsCKX4* to promote cytokinin degradation [12]. In strawberry (*Fragaria ananassa*), FveMYB117a directly binds to the promoters of *FveIPT2* and *FveCKX1* to regulate the expression of these two genes, which in turn regulates the content of cytokinin [13]. In addition, ZjWRKY23 and ZjWRKY40 promote fruit size in jujube by targeting and downregulating the expression of *CKX5* [14]. In Arabidopsis (*Arabidopsis thaliana*), the MADS-box transcription factor STK regulates fruit size by targeting *CKX7* and affecting the content of cytokinin [15].

As an important nutrient in plant growth and development, sugar not only serves as a carbon source but also acts as signal molecules that regulates metabolism, growth, development, and stress tolerance [16, 17]. Sorbitol is a sugar alcohol responsible for 60% to 80% photosynthetic products in apple and all other drupes in the Rosaceae family [18]. Recent studies reveal that sorbitol is a signaling regulator of stamen development, pollen tube growth, malate accumulation, and *Alternaria alternata* resistance in apple [19].

In this study, we find that cytokinin content is increased and the senescent rate of leaves is delayed in *A6PR* (*ALOSE-6-PHOSPHATE REDUCTASE*, a crucial enzyme in sorbitol synthesis) RNAi lines A4 and A10. Further transcriptome comparative analysis reveals the cytokinin oxidase *MdCKX7* is downregulated in A4 and A10 lines. And *MdCKX7* positively regulates leaf senescence by degrading cytokinins. Two plant-specific DOF transcription factors, *MdCDOF3* and *MdDOF3.6*, are co-expressed with *MdCKX7*. Biochemical experiments show that these two transcription factors bind directly to *MdCKX7* promoter and transcriptionally activate the expression of *MdCKX7* to promote leaf senescence. Interestingly, the expression of *MdCDOF3*, *MdDOF3.6,* and *MdCKX7* is induced by sorbitol. Taken together, this study reveals that sorbitol signaling activates *MdCDOF3*/*MdDOF3.6-MdCKX7* module and regulates leaf senescence in apple.

## Results

### Inhibition of sorbitol synthesis leads to slower leaf senescence and increased cytokinin content

In the previous study, we obtained two apple transgenic lines with defects in sorbitol synthesis (antisense inhibition of *ALOSE-6PHOSPHATE REDUCTASE A6PR*), which were named A4 and A10 (Supplemental Fig. 1). By observing the phenotype of apple transgenic seedlings, we found that A4 and A10 had less senescent leaves compared to the wild type. To better characterize the leaf phenotype of A4 and A10 plants, we defined the leaf development period as non-senescent stage (NS), early senescent stage (ES), and late senescent stage (LS). The results showed that the number of ES and LS leaves of 40-day-old A4 and A10 was only half of the wild type (WT) (Supplemental Fig. 2). To further prove the senescence rate of A4 and A10 leaves was slower than that of the wild type, we took samples every ten days and measured the leaf senescence index including the contents of chlorophyll, superoxide dismutase (SOD), and malondialdehyde (MDA). The results showed that the content of chlorophyll and SOD presented a downward trend with the increase of leaf age in both WT and transgenic lines. However, the chlorophyll and SOD contents in A4 and A10 decreased slower than the WT control. In addition, the MDA content in A4 and A10 leaves also decreased more slowly than the WT (Supplemental Fig. 3). These results indicate that A4 and A10 leaves had a slower rate of senescence than WT.

**Figure 1:**
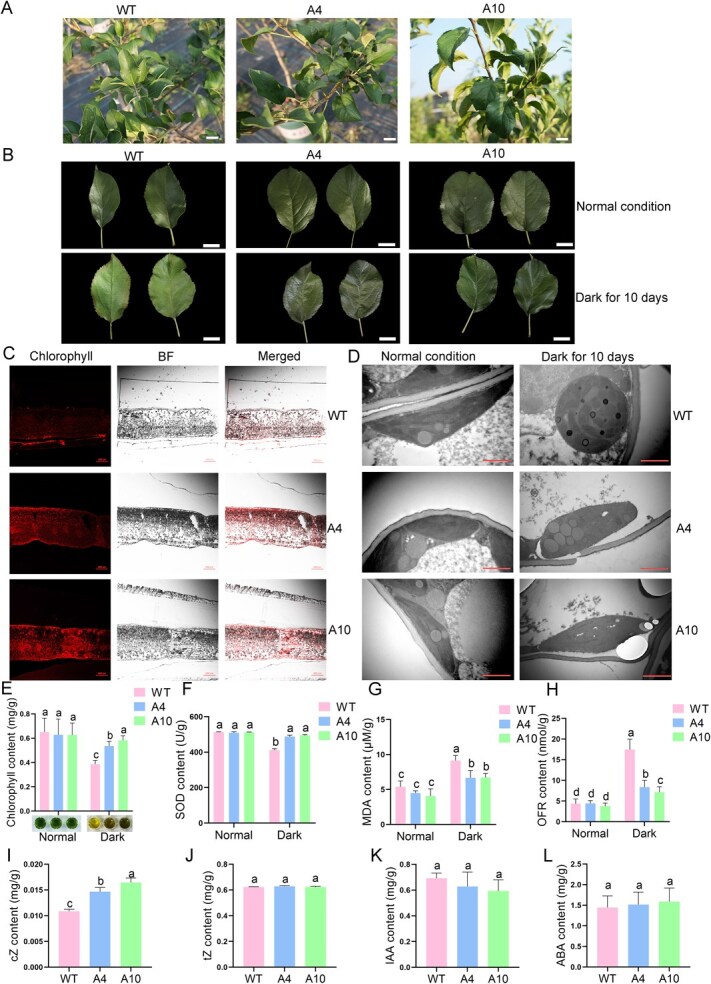
**The *A6PR* antisense lines showed elevated cZ content and delayed leaf senescence.** (A) Wild-type and *A6PR* antisense lines A4, A10 apple plants, Scale bars = 2 cm. (B) Wild-type, A4, A10 and leaves under normal condition and dark treatment. Wild-type leaves showed obvious senescence after dark treatment for 10 days. A4, A10 did not show significant senescence, Scale bars = 2 cm. (C) Chlorophyll auto-fluorescence at the interface of dark-treated leaves as observed by laser confocal microscopy. Scale bars = 100 μm. (D) The chloroplast morphology of 10 day dark treated wild-type and A4, A10 leaves was photographed by transmission electron microscopy (TEM) observation, Scale bars = 1 μm. (E-L) Chlorophyll (E), SOD (F), MDA (G), OFR (H), cZ (I), tZ (J), IAA (K), and ABA (L) content of wild-type and A4, A10 leaves under normal condition and dark treatment. SOD, Superoxide dismutase; MDA, Malondialdehyde; OFR, Superoxide anion; cZ, Cis-zeatin; tZ, Trans-zeatin; IAA, Indole acetic acid; ABA, Abscisic acid. Data are expressed as the mean ± SEM of three biological replicates. Different letters (a, b and c) indicate significant differences (*P* < 0.05) between different samples using Duncan’s multiple range test (MRT) after ANOVA.

We further used the leaves from the 4-year-old apple tree to explore whether A4 and A10 were less prone to senescence (Fig. 1A). Mature and healthy apple leaves were collected in late June for dark treatment. The leaf color from the wild type became yellow green after dark treatment for 10 days, indicating wild-type leaves were senescent. On the contrary, apple leaves from A4 and A10 still presented a deep green color after dark treatment, which was similar to those under normal condition (Fig. 1B). These results suggest that the senescent rate of leaves from A4 and A10 was slower than the WT. Due to the fact that leaf senescence was accompanied by the degradation of chloroplasts, we further observed chlorophyll auto-fluorescence from wild-type, A4 and A10 leaves under a fluorescence confocal microscope. The results indicated that the red fluorescence emitted by chlorophyll in dark-treated A4 and A10 leaves was significantly stronger than the WT (Fig. 1C). In addition, we observed and compared the structure of chloroplasts of the WT and two transgenic lines before and after dark treatment by transmission electron microscopy (TEM). The results showed that dark treatment resulted in the merging of membranes into large loose folds, the accumulation of plastids, the typical unapplied and disconnected stromal lamellipodia, and the swelling and anomalies in the lumens of vesicle-like tubules, which was a sign of chloroplast senescence. However, the chloroplasts of A4 and A10 leaves presented more slight abnormalities compared to the WT (Fig. 1D, Supplemental Fig. 4A). We also calculated the ratio of senescent chloroplasts to total chloroplasts within the 1500× field of view and found that both two *A6PR* antisense lines had a significantly lower proportion of senescent chloroplasts compared to the wild type (Supplemental Fig. 4B). Consistent with the above results, the chlorophyll content of A4 and A10 leaves had no significant difference compared to the wild type under normal condition. However, the chlorophyll content of A4 and A10 leaves was significantly higher than the WT after dark treatment (Fig. 1E). A series of physiological indices, including SOD activity, MDA content, and oxidative free radical (OFR) levels, were then determined to assess the degree of leaf senescence. The results showed that the SOD activities in A4 and A10 leaves were higher than the WT after dark treatment (Fig. 1F). On the contrary, the MDA content and OFR levels in A4 and A10 leaves were significantly lower than the WT (Fig. 1G and H). These results indicate that inhibition of sorbitol synthesis leads to slower leaf senescence.

As plant hormones play important roles in regulating leaf senescence, we wonder whether the contents of hormones have changed in A4 and A10 leaves. Based on the measurement, the cis-zeatin (cZ) content of A4 and A10 leaves was significantly higher than the WT (Fig. 1I), whereas the content of trans-zeatin (tZ), indole-3-acetic acid (IAA), and abscisic acid (ABA) had no obvious difference between the WT and transgenic lines (Fig. 1J–L). These results suggest that the delayed leaf senescence observed in A4 and A10 was likely attributable to the alteration in cytokinin content.

### The expression of *cytokinin oxidase MdCKX7* was significantly downregulated in A4 and A10 leaves

**Figure 2:**
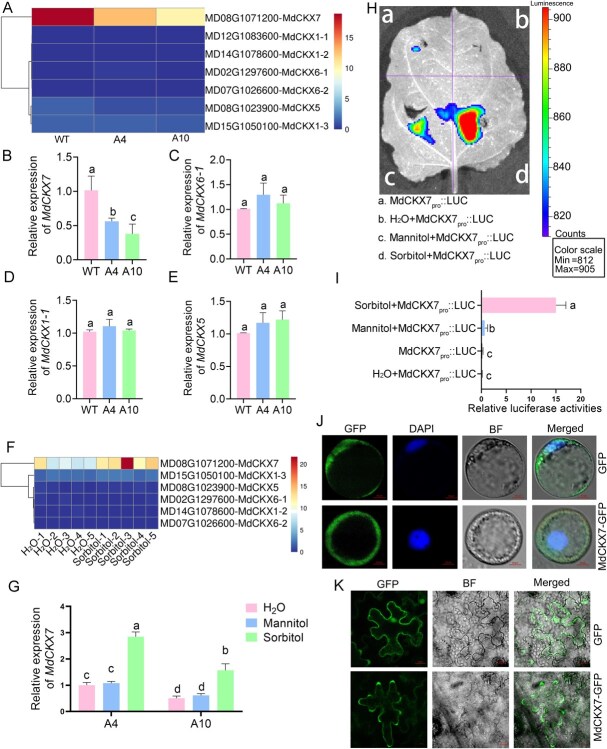
**The expression of sorbitol-responsive *MdCKX7* is downregulated in A4 and A10 leaves.** (A) Heat map analysis of *MdCKXs* based on RNA-Seq data from wild-type and A4, A10 leaves. (B-E) The expression levels of seven *MdCKX* genes based on RT-qPCR. Data are expressed as the mean ± SEM of three biological replicates. Different letters (a, b, and c) indicate significant differences (*P* < 0.05) between different samples using Duncan’s multiple range test (MRT) after ANOVA. (F) Heat map analysis of *MdCKXs* based on RNA-Seq data from H_2_O and sorbitol-fed A10 leaves. (G) The expression level of *MdCKX7* based on RT-qPCR in H_2_O, mannitol, and sorbitol-fed A4 and A10 leaves. Data are expressed as the mean ± SEM of three biological replicates. Different letters (a, b and c) indicate significant differences (*P* < 0.05) between different samples using Duncan’s multiple range test (MRT) after ANOVA. (H) Transient expression assays in tobacco leaves show that sorbitol induces the expression of *MdCKX7*. *MdCKX7pro::Luc* is used in this assay. The H_2_O and mannitol treatment are used as control. (I) Relative LUC activity at different injection sites. Luminescence is expressed as a ratio of the LUC to REN signals. Means and standard deviations were calculated from the results of three biological repetitions. Different letters (a, b and c) indicate significant differences (*P* < 0.05) using Duncan’s multiple range test (MRT) after ANOVA. (J) MdCKX7 is located in cytoplasm in apple protoplasts. DAPI is used to indicate nucleus. The GFP is used as control. Scale bars = 10 μm. (K) MdCKX7 is located in cytoplasm in Tobacco leaves. The GFP is used as control. Scale bars = 20 μm.

To further elucidate the reasons for the increase in cytokinin content and delayed senescent rate in A4 and A10 leaves, we analyzed the RNA-seq data of the WT, A4, and A10 leaves. A total of seven *CKX* genes were identified and only the expression of *MdCKX7* was differentially downregulated in A4 and A10 leaves compared to the WT (Fig. 2A). Further RT-qPCR results were also consistent with the RNA-seq analysis, indicating the elevated cytokinin content in A4 and A10 leaves was likely due to the downregulation of *MdCKX7* expression (Fig. 2B–E).

As sorbitol synthesis was dramatically obstructed in A4 and A10 leaves, we wondered whether the expression of *MdCKX7* was associated with sorbitol concentration. The results based on RNA-seq showed that the expression level of *MdCKX7* was significantly upregulated in apple leaves fed with 50 mM sorbitol compared to the leaves fed with H_2_O (Fig. 2F). Moreover, we fed A4 and A10 leaves with H_2_O, mannitol, and sorbitol (Supplemental Fig. 5A) and detected the expression level of *MdCKX7* before and after the feeding. The results showed that the expression level of *MdCKX7* under sorbitol treatment was significantly higher than that under H_2_O and mannitol treatment (Fig. 2G). These results indicate the expression of *MdCKX7* was activated by sorbitol. To further validate this conclusion, tobacco (*Nicotiana benthamiana*) leaves were coinjected with 50 mM sorbitol and *MdCKX7pro::*LUC recombinant plasmid, using H_2_O or mannitol as control. The results demonstrated that strong luminescent signals were detected in tobacco leaves coinjected with 50 mM sorbitol and *MdCKX7pro::*LUC recombinant plasmid. However, only very weak luminescent signals were detected in the negative control (Fig. 2H and I). To further validate the sorbitol-induced activation of the *MdCKX7* promoter in ‘Orin’ calli, we performed GUS reporter assays under 50 mM sorbitol treatment (using H₂O and mannitol as control). A GUS reporter system was generated by introducing the *MdCKX7_Pro_::1300* construct (harboring the GUS reporter gene) into ‘Orin’ calli. Histochemical analysis revealed significantly stronger GUS staining in sorbitol-treated transgenic calli compared to the control (Supplemental Fig. 6A), further confirming that the promoter activity of *MdCKX7* was enhanced by sorbitol treatment. These results suggest that the expression of *MdCKX7* is induced by sorbitol.

To validate the protein characteristics of MdCKX7, we first constructed an evolutionary tree using MdCKX7 and its homologous proteins in other species. The results showed that MdCKX7 is most homologous to CKX7 from *pyrus ussuriensis x pyrus communis* (Supplemental Fig. 7). The subcellular localization of MdCKX7 was then detected. We obtained ‘Orin’ calli overexpressing *MdCKX7* by transforming *35S*::*MdCKX7*-GFP recombinant plasmid into the calli. We further isolated protoplasts from obtained genetically stable apple calli and observed GFP signals under the laser confocal electron microscopy. The results showed that strong MdCKX7-GFP signals appeared in cytoplasm (Fig. 2J). Moreover, we also heterologously expressed *MdCKX7* with GFP tag in tobacco leaves, and GFP signals were located in the cytoplasm (Fig. 2K). These results indicate that MdCKX7 performs its biological functions within the cytoplasm.

### MdCKX7 accelerates leaf senescence by degrading cZ

To explore the function of MdCKX7, we first observed the phenotype of calli overexpressing *MdCKX7*. The results based on RT-qPCR confirmed that the expression level of *MdCKX7* in transgenic calli was significantly higher than the WT (Supplemental Fig. 8A). The cZ content was then determined by high-performance liquid chromatography (HPLC) assay. And the cZ content in calli overexpressing *MdCKX7* was significantly lower compared to that of WT (Supplemental Fig. 8B), suggesting that MdCKX7 possesses the function to degrade cZ.

**Figure 3:**
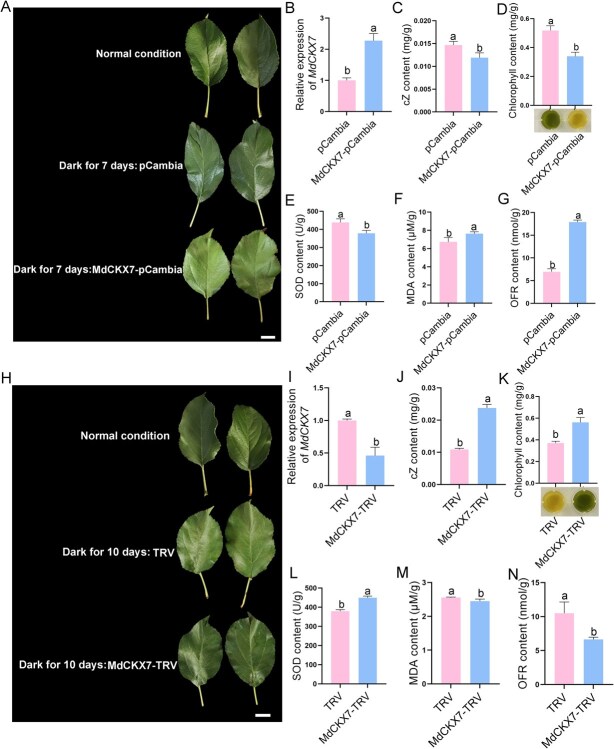
**MdCKX7 promotes leaf senescence by degrading cZ.** (A) Phenotype of apple leaves overexpressing *MdCKX7*. Scale bar = 2 cm. (B) Relative expression levels of *MdCKX7* in overexpressing apples based on RT-qPCR. Data are expressed as the mean ± SEM of three biological replicates. Different letters (a and b) indicate significant differences (*P* < 0.05) using Duncan’s multiple range test (MRT) after ANOVA. (C-G) The cZ (C), Chlorophyll (D), SOD (E), MDA (F), OFR (G) content of apple leaves overexpressing *MdCKX7* after a dark treatment for seven days, with pCambia as control. Data are expressed as the mean ± SEM of three biological replicates. Different letters (a, b and c) indicate significant differences (*P* < 0.05) using Duncan’s multiple range test (MRT) after ANOVA. (H) Phenotype of apple leaves silencing *MdCKX7*. Scale bar = 2 cm. (I) Relative expression levels of *MdCKX7* in apple leaves silencing *MdCKX7* based on RT-qPCR. Data are expressed as the mean ± SEM of three biological replicates. Different letters (a and b) indicate significant differences (*P* < 0.05) using Duncan’s multiple range test (MRT) after ANOVA. (J-N) The cZ (J), Chlorophyll (K), SOD (L), MDA (M), OFR (N) content of apple leaves silencing *MdCKX7* after a dark treatment for ten days, with TRV as a control. Data are expressed as the mean ± SEM of three biological replicates. Different letters (a, b, and c) indicate significant differences (*P* < 0.05) using Duncan’s multiple range test (MRT) after ANOVA.

To further investigate the role of *MdCKX7* in leaf senescence, the *35S::MdCKX7-*GFP recombinant plasmid were overexpressed in apple leaves, while the apple leaves expressing *35S::GFP* was used as the control. The expression level of *MdCKX7* in the overexpressed leaves was significantly higher than that of the control after a dark treatment for 7 days, confirming the successful overexpression (Fig. 3A and B). The contents of cZ, chlorophyll, and SOD in the leaves overexpressing *MdCKX7* were significantly lower compared to the control (Fig. 3C–E). On the contrary, the contents of MDA and OFR in the leaves overexpressing *MdCKX7* were significantly higher than the control (Fig. 3F and G). These results indicate that the overexpression of *MdCKX7* accelerates leaf senescence. Meanwhile, the expression level of *MdCKX7* was downregulated by viral transient silencing method (Fig. 3H and I). The decreased expression of *MdCKX7* leads to higher contents of cZ, chlorophyll and SOD (Fig. 3J–L). In addition, the inhibition of *MdCKX7* resulted in a reduction in both MDA and OFR contents (Fig. 3M and N). We further detected tZ content in transgenic leaves with transient overexpression and silencing of *MdCKX7*, showing no effect on tZ levels (Supplemental Fig. 9A and B). Taken together, these results suggest that MdCKX7 promotes leaf senescence by degrading cZ.

### *MdCDOF3* and *MdDOF3.6* respond to sorbitol signaling and accelerate leaf senescence by downregulating cZ content

**Figure 4:**
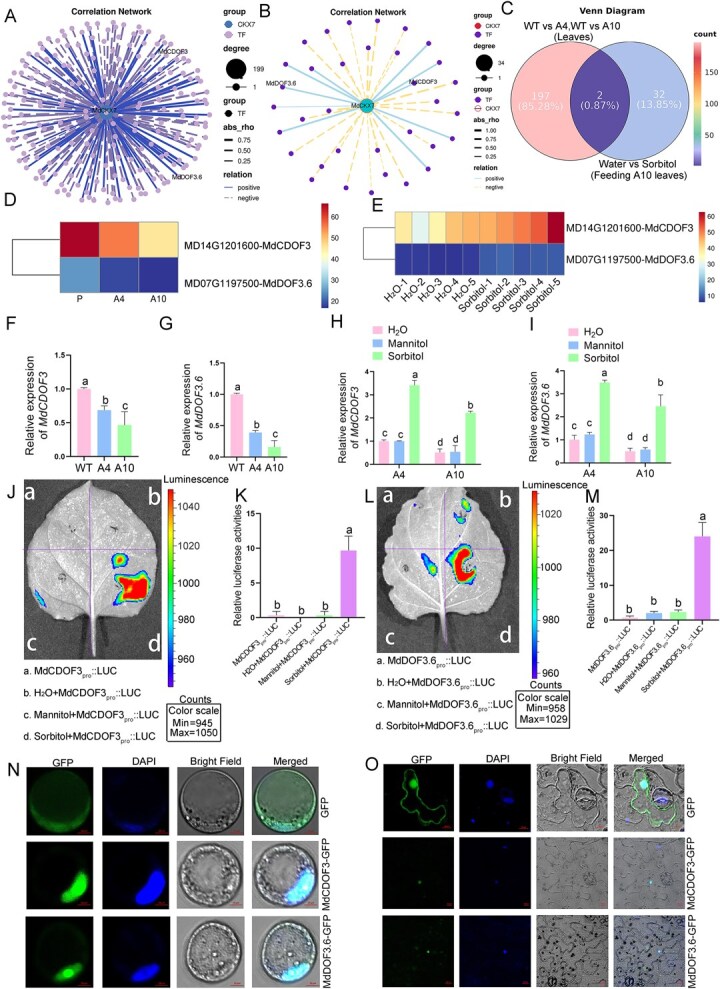
***MdCDOF3* and *MdDOF3.6* are co-expressed with *MdCKX7* and responsive to sorbitol signaling.** (A) Transcription factors associated with *MdCKX7* based on RNA-seq data of wild-type, A4 and A10 leaves. (B) Transcription factors associated with *MdCKX7* based on RNA-seq data of sorbitol-fed A10 leaves. (C) The intersection of (A) and (B). (D) The heat map of *MdCDOF3* and *MdDOF3.6* based on RNA-seq analysis of wild-type, A4 and A10 leaves. (E) The heat map of *MdCDOF3* and *MdDOF3.6* based on RNA-seq analysis of sorbitol-fed A10 leaves. (F-G) The expression levels of *MdCDOF*3 (F) and *MdDOF3.6* (G) in wild-type, A4 and A10 leaves based on RT-qPCR. Data are expressed as the mean ± SEM of three biological replicates. Different letters (a, b and c) indicate significant differences (*P* < 0.05) between different samples using Duncan’s multiple range test (MRT) after ANOVA. (H-I) The expression levels of *MdCDOF3* (H) and *MdDOF3.6* (I) in sorbitol-fed A4 and A10 leaves based on RT-qPCR, with H₂O and mannitol feeding as control. Data are expressed as the mean ± SEM of three biological replicates. Different letters (a, b and c) indicate significant differences (*P* < 0.05) between different samples using Duncan’s multiple range test (MRT) after ANOVA. (J-M) Transient expression assays in tobacco leaves show that sorbitol induces the expression of *MdCDOF3* (J) and *MdDOF3.6* (K). *MdCDOF3_pro_::Luc* and *MdDOF3.6_pro_::Luc* is used in this assay. The H_2_O and mannitol treatment are used as control. K and M indicate relative LUC activity at different injection sites shown in J and L, respectively. Luminescence is expressed as a ratio of the LUC to REN signals. Means and standard deviations were calculated from the results of and three biological repetitions. Different letters (a, b and c) indicate significant differences (*P* < 0.05) using Duncan’s multiple range test (MRT) after ANOVA. (N-O) MdCDOF3 (N) and MdDOF3.6 (O) are both located in nucleus in apple protoplasts. DAPI is used to indicate nucleus and the GFP is used as control. Scale bars = 10 μm.

The RNA-seq data from the leaves of the WT, A4, and A10 were utilized to identify a total of 199 candidate transcription factors that co-express with *MdCKX7* (Fig. 4A). Subsequently, we analyzed the RNA-seq data of A10 leaves that were fed with either 50 mM sorbitol or water and identified 34 transcription factors that coexpressed with *MdCKX7* (Fig. 4B). By taking the intersection of the 199 transcription factors and the aforementioned 34 transcription factors, we obtained two DOF transcription factors, namely *MdCDOF3* and *MdDOF3.6* (Fig. 4C). Both these two transcription factors were significantly downregulated in the leaves of A4 and A10 compared to the WT (Fig. 4D). Meanwhile, the expression levels of *MdCDOF3* and *MdDOF3.6* were markably higher under 50 mM sorbitol treatment compared to the water treatment (Fig. 4E). Further RT-qPCR results were also consistent with the RNA-seq analysis (Fig. 4F and G). Moreover, we fed A4 and A10 leaves with H_2_O, mannitol and sorbitol (Supplemental Fig. 5B) and detected the expression levels of *MdCDOF3* and *MdDOF3.6* before and after the feeding. The results showed that the expression levels of *MdCDOF3* and *MdDOF3.6* under sorbitol treatment were significantly higher than that under H_2_O and mannitol treatment. These results indicate that the expression of *MdCDOF3* and *MdDOF3.6* was activated by sorbitol (Fig. 4H and I). To further validate this conclusion, tobacco leaves were co-injected with 50 mM sorbitol and *MdCDOF3_pro_::LUC* or *MdDOF3.6_pro_::LUC* recombinant plasmid, using H_2_O or mannitol as control. The results demonstrated that strong luminescent signals were detected in tobacco leaves coinjected with 50 mM sorbitol and *MdCDOF3_pro_::LUC*/*MdDOF3.6_pro_::LUC* recombinant plasmid. However, only very weak luminescent signals were detected in the negative control (Fig. 4J–M). To further validate the promoter activity of *MdCDOF3* and *MdDOF3.6* under 50 mM sorbitol treatment (with H₂O and mannitol serving as controls), we conducted GUS reporter assays in ‘Orin’ calli. The *MdCDOF3_Pro_::1300* and *MdDOF3.6_Pro_::1300* constructs, harboring the GUS reporter gene, were introduced into ‘Orin’ calli to establish the GUS detection system. Following treatment with 50 mM sorbitol, H_2_O, or mannitol, histochemical analysis revealed that sorbitol-treated transgenic calli presented significantly more intense GUS staining compared to the control (Supplemental Fig. 6B and C). These results further indicate that the expression of *MdCDOF3* and *MdDOF3.6* was activated by sorbitol.

To validate the protein characteristics of MdCDOF3 and MdDOF3.6, we first constructed an evolutionary tree using MdCDOF3 and MdDOF3.6 and its homologous proteins in other species. The results showed that MdCDOF3 and MdDOF3.6 were most homologous to CDOF3 and DOF3.6 from *pyrus ussuriensis x pyrus communis* (Supplemental Fig. 10A and B). The subcellular localization of MdCDOF3 and MdDOF3.6 were then detected. We successfully obtained ‘Orin’ transgenic calli overexpressing *MdCDOF3-GFP* and *MdDOF3.6-GFP* by transforming the 35S::MdCDOF3-GFP and 35S::MdDOF3.6-GFP recombinant plasmids into apple calli, respectively. We isolated protoplasts from obtained genetically stable apple calli and observed GFP signals under the laser scanning confocal microscope. The results showed that strong MdCDOF3-GFP and MdDOF3.6-GFP signals appeared in nuclei (Fig. 4N). Moreover, we also heterologously expressed *MdCDOF3* and *MdDOF3.6* with GFP tag in tobacco leaves, and GFP signals were also located in the nuclei (Fig. 4O). These results indicate that MdCDOF3 and MdDOF3.6 perform their biological functions within the nucleus.

To further explore the function of *MdCDOF3* and *MdDOF3.6*, we first observed the phenotype of calli overexpressing *MdCDOF3* and *MdDOF3.6*. The results based on RT-qPCR assay confirmed that the expression levels of *MdCDOF3* and *MdDOF3.6* in transgenic calli were significantly higher than the WT (Supplemental Fig. 11A and C). The cZ content was then determined by HPLC assay. The cZ content in calli overexpressing *MdCDOF3* and *MdDOF3.6* was significantly lower compared to that of WT (Supplemental Fig. 11B and D), suggesting that both *MdCDOF3* and *MdDOF3.6* possess the function of decreasing cZ content.

**Figure 5:**
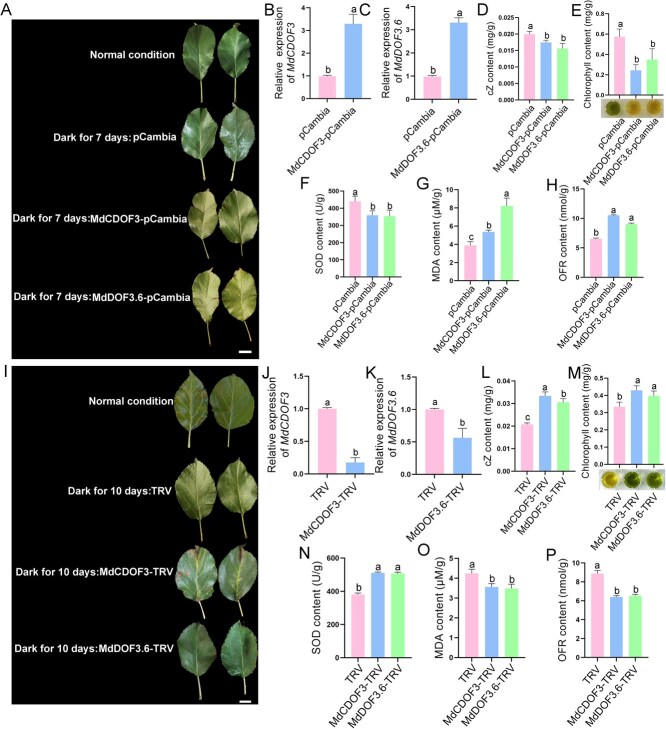
**MdCDOF3 and MdDOF3.6 promotes leaf senescence by decreasing cZ content.** (A) Phenotype of apple leaves overexpress *MdCDOF3* and *MdDOF3.6*. Scale bar = 2 cm. (B-C) Relative expression in levels of *MdCDOF3* and MdDOF3.6 in apple leaves overexpressing MdCDOF3 and MdDOF3.6 based on RT-qPCR. Data are expressed as the mean ± SEM of three biological replicates. Different letters (a and b) indicate significant differences (*P* < 0.05) using Duncan’s multiple range test (MRT) after ANOVA. (D-H) The cZ (D), Chlorophyll (E), SOD (F), MDA (G), OFR (H) content of apple leaves overexpressing *MdCDOF3* and *MdDOF3.6* after a dark treatment for seven days. Data are expressed as the mean ± SEM of three biological replicates. Different letters (a, b and c) indicate significant differences (*P* < 0.05) using Duncan’s multiple range test (MRT) after ANOVA. (I) Phenotype of apple leaves silencing *MdCDFO3* and *MdDOF3.6*. Scale bar = 2 cm. (I) Relative expression levels of *MdCDFO3* and *MdDOF3.6* in apple leaves silencing *MdCDFO3* and *MdDOF3.6* based on RT-qPCR. Data are expressed as the mean ± SEM of three biological replicates. Different letters (a and b) indicate significant differences (*P* < 0.05) using Duncan’s multiple range test (MRT) after ANOVA. (J-N) The cZ (J), Chlorophyll (K), SOD (L), MDA (M), OFR (N) content of apple leaves silencing *MdCDFO3* and *MdDOF3.6* after a dark treatment for ten days. Data are expressed as the mean ± SEM of three biological replicates. Different letters (a, b, and c) indicate significant differences (*P* < 0.05) using Duncan’s multiple range test (MRT) after ANOVA.

To further investigate the role of *MdCDOF3* and *MdDOF3.6* in leaf senescence, the *35S::MdCDOF3*-GFP and *35S::MdDOF3.6*-GFP recombinant plasmids were transformed into apple leaves to overexpress *MdCDOF3* and *MdDOF3.6*, respectively. And the apple leaves expressing *35S::GFP* were used as the control. The results obtained from RT-qPCR assays indicated that the expression of both *MdCDOF3* and *MdDOF3.6* in overexpressed leaves treated in the dark for seven days was significantly higher compared to the control. (Fig. 5A–C). The contents of cZ, chlorophyll, and SOD in the leaves overexpressing *MdCDOF3* and *MdDOF3.6* were significantly lower than that of the control (Fig. 5D–F). On the contrary, the MDA and OFR contents in the leaves overexpressing *MdCDOF3* and *MdDOF3.6* were significantly higher compared to the control (Fig. 5G and H). These results indicate the overexpression of *MdCDOF3* and *MdDOF3.6* accelerates leaf senescence. Meanwhile, the expression of *MdCDOF3* and *MdDOF3.6* was downregulated by the viral transient silencing method (Fig. 5I–K). The results revealed that the decreased expression of *MdCDOF3* and *MdDOF3.6* led to higher cZ, chlorophyll and SOD contents (Fig. 5L–N). In addition, the inhibition of *MdCDOF3* and *MdDOF3.6* resulted in lower MDA and OFR contents (Fig. 5O and P). We further detected the tZ content in apple leaves overexpressing and silencing *MdCDOF3* and *MdDOF3.6*, respectively. The results showed that *MdCDOF3* and *MdDOF3.6* had no effect on tZ levels (Supplemental Fig. 12). Taken together, these results suggest that both *MdCDOF3* and *MdDOF3.6* promote leaf senescence by decreasing cZ content.

### MdCDOF3 and MdDOF3.6 directly bind *MdCKX7* promoter and activate its expression

**Figure 6:**
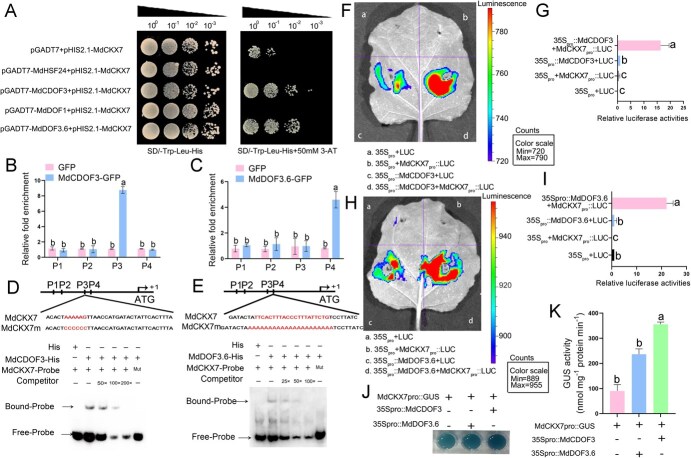
**MdCDOF3 and MdDOF3.6 bind to *MdCKX7* promoter and activate its transcription.** (A) Yeast one-hybridization (Y1H) assays showed that MdCDOF3 and MdDOF3.6 interacted with *MdCKX7* promoter. The 2200 bp upstream *MdCKX7* promoter sequence of ATG is used for detection. (B-C) The relative levels of *MdCKX7* promoter fragment enriched by MdCDOF3 (B) and MdDOF3.6 (C) detected by chromatin immunoprecipitation (ChIP)-qPCR. Data are expressed as the mean ± SEM of three biological replicates. Different letters (a and b) indicate significant differences (*P* < 0.05) using Duncan’s multiple range test (MRT) after ANOVA. (D–E) Electrophoretic shift assay (EMSA) determination of the interaction of MdCDOF3 (D) and MdDOF3.6 (E) with labeled DNA Probes in the *MdCKX7* promoter. (F-I) Luciferase assays showed that MdCDOF3 (F and G) and MdDOF3.6 (H and I) activated *MdCKX7* expression in Tobacco leaves by transient injection. Data are expressed as the mean ± SEM of three biological replicates. Different letters (a, b, and c) indicate significant differences (*P* < 0.05) using Duncan’s multiple range test (MRT) after ANOVA. (J-K) GUS staining and activity of apple calli co-expressing different combinations of recombinant plasmids including *MdCKX7_pro_*::GUS, *35S_pro_*::MdCDOF3 + *MdCKX7_pro_*::GUS, and *35S_pro_*::MdDOF3.6 + *MdCKX7_pro_*::GUS. Data are expressed as the mean ± SEM of three biological replicates. Different letters (a, b and c) indicate significant differences (*P* < 0.05) using Duncan’s multiple range test (MRT) after ANOVA.

Given the co-expression of *MdCDOF3* and *MdDOF3.6* with *MdCKX7*, along with their positive roles in leaf senescence, we speculated that MdCDOF3 and MdDOF3.6 may promote leaf senescence through transcriptional regulation of *MdCKX7*. To validate this speculation, we first detected the *in vitro* binding of MdCDOF3 and MdDOF3.6 with the *MdCKX7* promoter by yeast one-hybrid (Y1H) assay. The promoter region of *MdCKX7* was fused to pHIS2 vector, and the coding sequences of *MdCDOF3* and *MdDOF3.6* were fused to the activation domain (AD) of pGADT7, respectively. The yeast cells co-expressing *MdCKX7pro::pHIS* with MdCDOF3-AD or MdDOF3.6-AD could grow normally on SD/−Leu/−Trp/-His+3-AT^50 mM^ plates, whereas no growth was seen in yeast cells co-expressing *MdCKX7pro::pHIS* with pGADT7 empty vector (Fig. 6A). These results indicate MdCDOF3-AD and MdDOF3.6-AD interact with *MdCKX7* promoter in yeast cells.

DOF transcription factors are known to regulate downstream target genes by recognizing *cis*-acting elements (5′-AAAAAG-3′) in the promoters of target genes [12]. We analyzed the *MdCKX7* promoter and obtained four potential binding sites of DOF transcription factors, namely P1, P2, P3, and P4. Chromatin immunoprecipitation PCR (ChIP-PCR) assay was performed to identify the binding regions of *MdCKX7* promoter using *35S*::MdCDOF3-GFP and *35S*::MdDOF3.6-GFP transgenic apple calli. The results showed that there was about 9-fold enrichment in the P3 region of *MdCKX7* promoter in *35S*::MdCDOF3-GFP transgenic calli (Fig. 6B). And there was approximately 5-fold enrichment in the P4 region of *MdCKX7* promoter in *35S*::MdDOF3.6-GFP transgenic calli (Fig. 6C). However, no obvious enrichment was observed in the other regions of the *MdCKX7* promoter compared with the control. These results indicate that both MdCDOF3 and MdDOF3.6 bind to the *MdCKX7* promoter *in vivo*.

To further determine the direct binding of MdCDOF3 and MdDOF3.6 with the *MdCKX7* promoter, we performed an electrophoretic mobility shift assay (EMSA) using purified recombinant His-MdCDOF3 and His-MdDOF3.6 fusion proteins probed with potential binding regions of DOF transcription factors in the *MdCKX7* promoter. Specific blocking shift of DNA probe-MdCDOF3 was detected when the DOF binding site core sequence (AAAAG)-containing oligonucleotide in the P3 region of *MdCKX7* promoter was used as the labeled probe (Fig. 6D). For MdCDOF3.6, specific blocking shift of DNA probe-MdDOF3.6 was detected when the DOF binding site core sequence (TTCACTTTACCCTTTATTCTG)-containing oligonucleotide in the P4 region of *MdCKX7* promoter was used as the labeled probe (Fig. 6E). This specific blocking shift was diminished when biotin-labeled probes were added and disappeared when the DNA probes were mutated (Fig. 6D and E). These results demonstrate that MdCDOF3 and MdDOF3.6 interact with the P3 and P4 regions of *MdCKX7* promoter, respectively.

To investigate whether MdCDOF3, MdDOF3.6 could transcriptionally activate the expression of *MdCKX7*, we coinjected the construct of *MdCKX7* fused with reporter gene luciferase (*MdCKX7pro*::Luc) with *35Spro*::MdCDOF3 and *35Spro*::MdDOF3.6, respectively, into tobacco leaves for expression. Tobacco leaves coinjected with *35Spro*::MdCDOF3 and *MdCKX7pro*::Luc, or *35Spro*::MdDOF3.6 and *MdCKX7pro*::Luc showed much stronger fluorescence signal compared to the control, indicating that both MdCDOF3 and MdDOF3.6 could enhance the expression of *MdCKX7* (Fig. 6F–I). We further validated the transcriptional activation of MdCDOF3 and MdDOF3.6 on *MdCKX7* using apple calli. The constructs *35S*::MdCDOF3 or *35S*::MdDOF3.6 were co-expressed with *MdCKX7_Pro_*::1300 (containing the GUS reporter) in apple calli to set up a GUS reporter gene assay system. We found that the transgenic apple calli co-expressing *MdCKX7_Pro_*::1300 + *35S*::MdCDOF3 and *MdCKX7_Pro_*::1300 + *35S*::MdDOF3.6 presented GUS staining deeper compared to the control (Fig. 6J). Consistent with the above results, the GUS activity of apple calli co-expressing *MdCKX7_Pro_*::1300 + *35S*::MdCDOF3 or *MdCKX7_Pro_*::1300 + *35S*::MdDOF3.6 was much higher than the control (Fig. 6K). These results indicate that MdCDOF3 and MdDOF3.6 transcriptionally activate the expression of *MdCKX7* by directly interacting with *MdCKX7* promoter.

### MdCDOF3 and MdDOF3.6 act upstream of *MdCKX7* to promote leaf senescence

**Figure 7:**
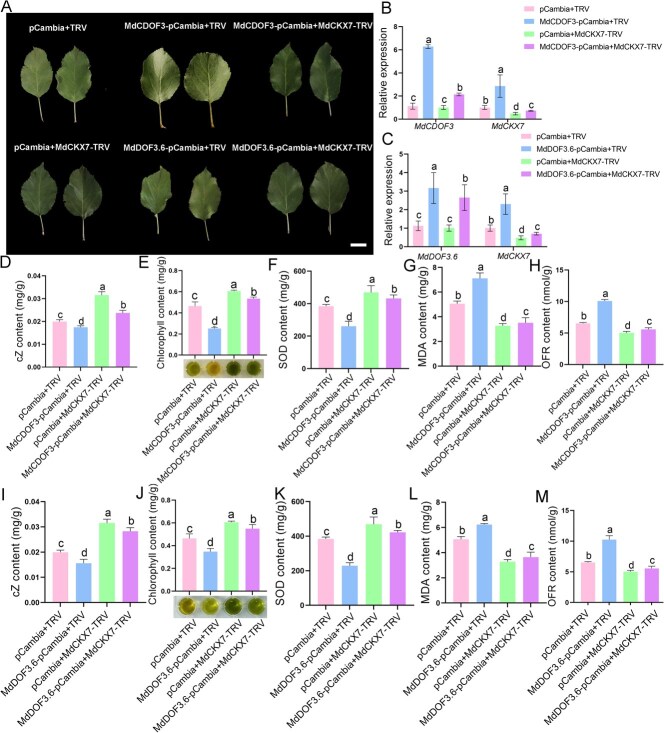
**MdCDOF3 and MdDOF3.6 act upstream of *MdCKX7* to promote leaf senescence.** (A) The phenotype of apple leaves co-expressing different combinations of recombinant plasmids including pCambia+TRV, MdCDOF3-pCambia+TRV, MdCDOF3-pCambia+ MdCKX7-TRV, pCambia+MdCKX7-TRV, MdDOF3.6-pCambia+TRV, MdDOF3.6-pCambia+ MdCKX7-TRV. The apples leaves were under dark treatment for 10 days. Scale bars = 2 cm. (B) Relative expression levels of *MdCDOF3* and *MdCKX7* in apple leaves co-expressing pCambia+TRV, MdCDOF3-pCambia+TRV, MdCDOF3-pCambia+ MdCKX7-TRV, pCambia+MdCKX7-TRV. Data are expressed as the mean ± SEM of three biological replicates. Different letters (a, b, c, and d) indicate significant differences (*P* < 0.05) using Duncan’s multiple range test (MRT) after ANOVA. (C) Relative expression levels of *MdDOF3.6* and *MdCKX7* in apple leaves co-expressing pCambia+TRV, MdDOF3.6-pCambia+TRV, MdDOF3.6-pCambia+MdCKX7-TRV, pCambia+MdCKX7-TRV. Data are expressed as the mean ± SEM of n = 3 biological replicates. Different letters (a, b, c and d) indicate significant differences (*P* < 0.05) using Duncan’s multiple range test (MRT) after ANOVA. (D-H) The cZ (D), Chlorophyll (E), SOD (F), MDA (G), OFR (H) content of apple leaves co-expressing pCambia+TRV, MdCDOF3-pCambia+TRV, MdCDOF3-pCambia+ MdCKX7-TRV, pCambia+MdCKX7-TRV. Data are expressed as the mean ± SEM of n = 3 biological replicates. Different letters (a, b, c and d) indicate significant differences (*P* < 0.05) using Duncan’s multiple range test (MRT) after ANOVA. (I-M) The cZ (I), Chlorophyll (J), SOD (K), MDA (L), OFR (M) content of apple leaves co-expressing pCambia+TRV, MdDOF3.6-pCambia+TRV, MdDOF3.6-pCambia+ MdCKX7-TRV, pCambia+MdCKX7-TRV. Data are expressed as the mean ± SEM of three biological replicates. Different letters (a, b, c, and d) indicate significant differences (*P* < 0.05) using Duncan’s multiple range test (MRT) after ANOVA.

Given that both of these two DOF transcription factors (MdCDOF3 and MdDOF3.6) and MdCKX7 play crucial roles in leaf senescence, and that MdCDOF3 and MdDOF3.6 transcriptionally activate the expression of *MdCKX7*, we speculate that *MdCDOF3* and *MdDOF3.6* act upstream of *MdCKX7* in promoting leaf senescence. To verify this speculation, we manipulated the expression levels of these three genes using the viral vector-based transformation method in apple leaves. Three viral constructs MdCDOF3-pCambia, MdDOF3.6-pCambia, and MdCKX7-TRV were created and infiltrated into apple leaves individually or as combination, with the empty vectors as the control (Fig. 7A). The results showed that when *MdCDOF3* or *MdDOF3.6* were overexpressed individually, the expression of *MdCKX7* was elevated. And when *MdCDOF3* or *MdDOF3.6* were overexpressed separately and meanwhile *MdCKX7* was silenced, the expression level of *MdCKX7* in this case was higher than that in the case of silencing *MdCKX7* individually (Fig. 7B and C). These results indicate that *MdCDOF3* and *MdDOF3.6* act upstream of *MdCKX7* in apple leaves. Subsequently, we analyzed the phenotype of the transformed apple leaves. The results showed that the cZ content in apple leaves coinfected with MdCDOF3-pCambia and MdCKX7-TRV was significantly higher than the apple leaves overexpressing *MdCDOF3* alone, but significantly lower than the apple leaves silencing *MdCKX7* alone (Fig. 7D). In addition, the content of chlorophyll and SOD showed similar trends of change with the cZ content (Fig. 7E and F). On the contrary, the content of MDA and OFR presented reversed trends of change compared to that of the cZ (Fig. 7G and H). Moreover, the effects of MdDOF3.6 were similar to that of MdCDOF3 (Fig. 7I–M). Taking together, these results suggest that MdCDOF3 and MdDOF3.6 positively regulate the degradation of cZ by transcriptionally activating the expression of *MdCKX7*, thereby promoting leaf senescence.

## Discussion

Sorbitol, a common sugar alcohol, is the major photosynthetic product in apples and other drupes of the Rosaceae family, accounting for 60% to 80% of leaf photosynthesis production and phloem transport [18, 20]. Meanwhile, sorbitol is not only used as a nutrient source for plant growth and life activities but also serves as a signaling substance for fruit and stem tip carbohydrate metabolism [20, 21], as well as for flower development and pollen tube growth [19, 22]. In this study, we found that sorbitol negatively regulates the cZ content in apple leaves by activating the expression of two transcription factors (*MdCDOF3* and *MdDOF3.6*) and a cytokinin oxidase (*MdCKX7*). Moreover, both MdCDOF3 and MdDOF3.6 activate the transcription of *MdCKX7* by binding to its promoter, which in turn accelerates the degradation of cZ, thereby promoting leaf senescence through the cytokinin pathway.

**Figure 8:**
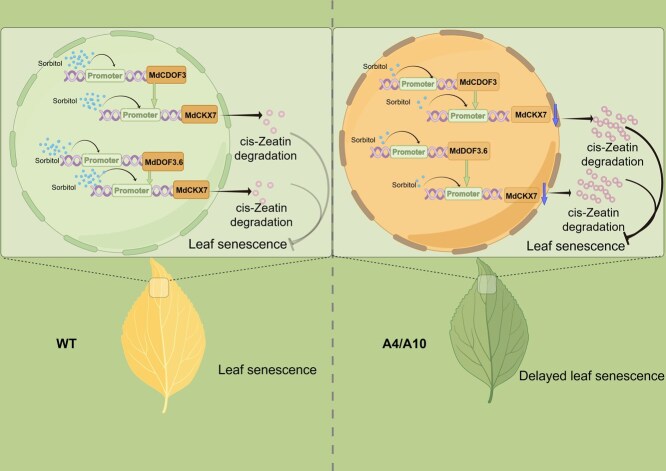
**A pattern diagram of MdCDOF3/MdDOF3.6-MdCKX7 mediated leaf senescence.** In the wild type, sorbitol synthesis proceeds normally, prompting the MdCDOF3/MdDOF3.6-MdCKX7 regulatory module to respond to sorbitol signaling and facilitate the degradation of cytokinin, ultimately resulting in leaf senescence. However, in the A4 and A10 varieties, the antisense expression of *A6PR* inhibits sorbitol synthesis in transgenic apple leaves, leading to the downregulation of the MdCDOF3/MdDOF3.6-MdCKX7 module. Consequently, the degradation of cytokinin (cZ) is impeded, causing an accumulation of cZ. As a result, the senescence process in the leaves of A4 and A10 are delayed.

A slower rate of leaf senescence is exhibited in the *A6PR* anti-sense lines (A4, A10) (Fig. 1B). This phenotype may be attributed to the increase of cytokinin content (Fig. 1I). Cytokinin oxidase 7, a key enzyme catalyzing the degradation of cytokinin, plays significant roles in regulating cytokinin content. We identify differentially expressed genes participating in the cytokinin pathway between the WT and *A6PR* antisense lines and find that *MdCKX7* was significantly downregulated in *A6PR* antisense lines. Interestingly, the response of *MdCKX7* to exogenous sorbitol could be detected by both RT-qPCR in apple leaves (Fig. 2G–H) and transient dual luciferase assay in tobacco leaves (Fig. 2I–J). Overexpression of *MdCKX7* in the WT shows a faster rate of leaf senescence (Fig. 3A), whereas transient silencing of *MdCKX7* showed a slower rate of leaf senescence compared to the control (Fig. 3H). These results demonstrate that the increase in cZ caused by the decrease in sorbitol content is mainly due to the downregulation of *MdCKX7*, leading to the delayed senescence in A4 and A10. However, the mechanism of how cytokinins retard leaf senescence in *A6PR* antisense lines is still unclear. It is currently reported that cytokinins retarding leaf senescence may be due to the fact that cytokinin signaling genes such as *HKs*, *B-type RRs,* and *CRFs* are closely associated with the onset of leaf senescence [23]. In addition, it has been shown that the accumulation of cytokinin content leads to elevated sucrose content [24], and in our previous study, we find that the *A6PR* antisense lines present significantly higher sucrose content [18], indicating cytokinins and sugar may act as a feedback regulatory loop to participate leaf senescence. We also found that the sorbitol content in apple leaves was significantly lower in normal leaves than that in the senescent leaves (Supplemental Fig. 13). This may be due to the fact that leaf senescence may be a stress factor for plant growth, which in turn leads to sorbitol accumulation [25]. Further sorbitol treatment assays demonstrated that sorbitol accelerated the senescence of wild-type leaves, as indicated by decreased chlorophyll and SOD content and increased MDA and OFR content (Supplemental Fig. 14), further supporting the idea that sorbitol promotes leaf senescence. We speculate that excessive sorbitol accumulation affects carbon metabolism and other important physiological processes in the plant, leading to metabolic imbalance and ultimately promoting leaf senescence [26].

DOF transcription factors, as plant species-specific regulators, have been found to play significant roles in leaf senescence. Overexpression of *MdCDOF3* and *MdDOF3.6* in apple was observed to promote leaf senescence (Fig. 5 A–N), which is similar with the role of *CDOF4* in Arabidopsis. The Arabidopsis *CDOF4* has been reported to promote leaf senescence and floral organ abscission through the modulation ABA and reactive oxygen species pathways [27]. Additionally, the MYC2-Dof2.1-MYC2 pathway promotes leaf senescence in Arabidopsis [28]. Previous studies suggest that the regulation of leaf senescence by DOF transcription factors is initiated by hormones, such as ABA and JA, which are known to promote leaf senescence [28, 29]. In our work, we reveal that MdCDOF3 and MdDOF3.6 bind to the *MdCKX7* promoter and activate its expression, thereby promoting the degradation of cytokinins and leaf senescence. This is confirmed through *in vitro* assays including Y1H, EMSA, and the *in vivo* ChIP-qPCR assays (Fig. 6A–F). Further LUC and GUS analyses indicate MdCDOF3 and MdDOF3.6 transcriptionally activate the expression of *MdCKX7* (Fig. 6J–L). Furthermore, we find that *MdCDOF3* and *MdDOF3.6* also respond to sorbitol signaling, which is similar to *MdCKX7*. Therefore, we speculate the decreased sorbitol synthesis leads to reduced expression levels of *MdCDOF3*, *MdDOF3.6,* and *MdCKX7*. And the downregulation of these three genes results in the accumulation of cytokinin, causing a delay in leaf senescence in the *A6PR* antisense lines compared to the wild type.

Finally, we propose a working model: In the wild type, sorbitol synthesis occurs normally. The MdCDOF3/MdDOF3.6-MdCKX7 module responds to sorbitol signaling and promotes the degradation of cytokinin, ultimately leading to leaf senescence. However, in A4 and A10 transgenic apple leaves, the antisense expression of *A6PR* inhibits sorbitol synthesis, resulting in the downregulation of the MdCDOF3/MdDOF3.6-MdCKX7 module. Consequently, the degradation of cZ is inhibited, causing an increase in its content. This, in turn, delays the senescence of A4 and A10 leaves (Fig. 8). These findings indicate that sorbitol serves as a signaling molecule in regulating the cytokinin pathway and further supports the notion that cytokinins can delay leaf senescence.

## Materials and methods

### Plant materials and growth condition

In this study, two transgenic lines were used to repress *A6PR* (A4 and A10) via antisense repression, and untransformed wild type (WT) ‘Greensleeves’ apples were used as the control. The expression level of *A6PR* in A4 was approximately 50% of that observed in the wild type (WT), while the expression level of *A6PR* in A10 was 50% of that in A4 [18].

Under natural conditions in the city of Tai’an, Shandong Province, 4-year-old trees WT, A4 and A10 on M26 rootstocks were grown in 20-liter pots with a culture solution containing 1 part sand: 2 parts cow dung. All trees received standard horticultural and insect control measures. Healthy mature leaves were collected in late June, frozen in liquid nitrogen, stored at −80°C.

‘Greensleeves’ apple variety *in vitro* tissue cultures were grown on Murashige and Skoog (MS) medium under 16-hour light/8-hour dark (light/dark) long day conditions with the addition of 0.1 mg/L IAA and 0.3 mg/L of 6-BA and incubated at room temperature with succession culture every 6 weeks.

Wild-type ‘Orin’ apple calli were cultured on MS with the addition of 1.5 mg/L 6-BA and 0.5 mg/L IAA medium at 25°C in the dark. Three repeated apple tissue cultures were performed every two weeks before being used for genetic transformation and other experiments.

### Leaf senescence assays

The isolated apple leaves were wrapped with a sponge moistened with water to cover the petiole and laid flat in a petri dish, which was placed in a dark incubator at 24°C for phenotypic observation, and the chlorophyll, SOD, MDA, and OFR contents were determined after recording the phenotypes.

### Feeding leaves with exogenous sorbitol

Fully expanded leaves of WT, A4, and A10 were fed with mannitol, sorbitol, and water, respectively, via a transpiration stream. These leaves were clipped from branches and brought to the laboratory at 7:30 a.m. Leaves were randomly assigned to be treated with 50 mM mannitol, 50 mM sorbitol, and water. Each treatment was replicated three times, and nine leaves were placed in each replicate. Leaves were placed in petri dishes with petioles held in filter paper, which was soaked in 50 mM mannitol, 50 mM sorbitol, and water. The room temperature photosynthetically active radiation was about 100 μmol photons m^−2^∙s^−1^. Leaf samples were collected at 0, and 6 h after initiation of sugar feeding, frozen in liquid nitrogen and stored at −80°C for later use [30].

### Hormone extraction

The leaves were ground with liquid nitrogen, 0.1 g sample was weighed in a 10 mL centrifuge tube and extracted with 80% methanol (containing 1% glacial acetic acid) and incubated in the extracting solution for 16 hours. And 2 mL extracting solution was added every 4 hours. After 16 hours, the sample was centrifuged at 4°C for 10 minutes at 6000 r/min, and the supernatant was taken in a new centrifuge tube.

### Determination of plant hormones

The determination was carried out on a Shimadzu ultrahigh liquid chromatograph with the mobile phase of chromatographic methanol (national medicine): ultrapure water (containing 1% glacial acetic acid) = 45:55. The extracted supernatant was passed through a 0.22-μm organic filter, and the filtrate was put into a 1.5-mL injection vial with a flow rate of 1 mL/min, an injection volume of 10 μL, and the time programmed as 35 minutes, with the detection wavelength of 254 nm.

### Extraction and determination of chlorophyll

Chlorophyll content was measured as previously described [29]. Chlorophyll was extracted by cutting plant leaves into small pieces and immersing them in 95% ethanol for 24 hours at 4°C. Absorbance was measured at 649 and 665 nm using a spectrophotometer (SOPTOP UV2800S, Shanghai, China).

### Determination of peroxide content and enzyme activity in membranes

Weighing 0.1 g apple leaf samples, the specific method was used to determine MDA, SOD, and OFR content by the MDA assay kit (Suzhou Kemin Biotechnology Co., Ltd, Suzhou, China), the SOD assay kit (NBT method) (Suzhou Kemin Biotechnology Co., Ltd, Suzhou, China), and the OFR detection kit (Suzhou Kemin Biotechnology Co., Ltd, Suzhou, China), respectively.

### Protoplast isolation

Weigh 2.5 to 3 g apple calli with appropriate growth status in 50 mL centrifuge tubes, enzymatically dissolve the cell wall through cell wall enzyme solution configured with pectinase, and then static filtration with 200 mesh nylon cloth at 4°C, collect the protoplasts that sink to the bottom of the centrifuge tubes, and store them at 4°C, as previously described by [31].

### RNA extraction, RT-PCR and qRT-PCR assays

RNA extraction, RT-PCR and qRT-PCR assays were performed with the methods as described by [32].

### Plasmid construction and genetic transformation

The coding sequences of *MdCKX7*, *MdCDOF3*, *MdDOF3.6* were amplified and inserted into pCambia::GFP vector to overexpress *MdCKX7*, *MdCDOF3*, *MdDOF3.6* under *35S* promoter. These vectors were genetically transformed into ‘Orin’ calli using *Agrobacterium* (LBA4404)-mediated transformation as previously described by [32].

### ChIP-qPCR assay

ChIP-qPCR analysis was performed using 35S::MdCDOF3-GFP, 35S::MdDOF3.6-GFP and 35S::GFP transgenic apple calli. An anti-GFP antibody (Beyotime, Shanghai, China) was used for ChIP as described by [33].

### Y1H assay

The coding sequences of *MdCDOF3* and *MdDOF3.6* were ligated into the pGAD424 vector (Clontech). The *MdCKX7* promoter was cloned into the pHIS2.1 vector (Clontech). Y1H assay was performed as previously described [34].

### EMSA assay

EMSA was performed according to a previous study [35]. The coding sequences of *MdCDOF3* and *MdDOF3.6* were cloned into the expression vector pET-30a-c. MdCDOF3-His, MdDOF3.6-His recombinant proteins were expressed in *E. coli* strain BL21 and the purification of recombinant proteins depended on specific binding of a histidine tag (His-tag) to immobilized metal ions (nickel) (Thermo Scientific, San Jose, CA, USA) [36]. Oligonucleotide probes for the *MdCKX7* promoter were labeled using the EMSA Probe Biotin Labeling Kit (Beyotime, Shanghai, China) according to the manufacturer’s instructions. The steps are as previously described [37].

### GUS analysis

Reporter gene constructs containing the *MdCKX7* promoter sequence were prepared as previously described [38].

### Transient dual-luciferase assays

The *MdCKX7* promoter region with a 2200 bp upstream of the starting codon was cloned into the pGreenII 0800-LUC dual reporter vector, and *MdCDOF3* and *MdDOF3.6* CDS were cloned into the pGreenll 62-SK vector as effectors. Recombinant plasmids were transformed into *Nicotiana benthamiana* leaves using *Agrobacterium* (LBA4404)-mediated transformation techniques [39]. Tobacco plants were incubated at 25°C for 2 to 3 days [40]. The ratio of firefly (*Photinus pyralis* to *Renilla reniformis*) LUC activity was used as an indicator of the transcriptional efficiency of MdCDOF3, MdDOF3.6 on the *MdCKX7* promoter.

### Construction and transient expression of apple leaf viral vectors

Using apple leaf cDNA as template, PCR amplified 200 to 300 bp fragments of specific regions of *MdCKX7*, *MdCDOF3,* and *MdDOF3.6* cDNAs, and constructed antisense viral vectors, which were used as templates for the expression of *MdCKX7*, *MdCDOF3,* and *MdDOF3.6* cDNA as previously described [37].

### Chlorophyll fluorescence observation

Chlorophyll fluorescence of leaf sections was analyzed as described according to a previous study [41] and observed using a LSCM880 laser confocal microscope from Zeiss, Germany, with a laser excitation of 514 nm. Chlorophyll was detected using a long-pass filter at 650 nm.

### TEM observation

Transmission electron microscopic studies of apple leaves followed the standard method described by a previous study [42]. The samples were observed through a JEOL 100CX transmission electron microscope (Jeol, Peabody, MA, USA).

### Statistical analysis

All experiments were performed at least in triplicate. Numbers represent the mean ± SD of three biological replicates. Statistical analyses of the data were conducted using analysis of variance (ANOVA), followed by Duncan’s multiple range test (MRT) for the experiments.

#### Data accessibility

Sequence data from this article can be found in the Genome Database for Rosaceae (https://www.rosaceae.org/) under accession numbers *MdCKX7* (MD08G1071200), *MdCDOF3* (MD14G1201600), *MdDOF3.6* (MD07G1197500).

## Supplementary Material

Web_Material_uhaf120

## Data Availability

All data generated or analyzed during this study are provided in the article and its supplementary data files.
