# Supplementary material for: Two DNA-binding One Zinc Finger transcription factors, MdCDOF3 and MdDOF3.6, accelerate leaf senescence by activating cytokinin oxidase MdCKX7 in response to sorbitol signaling in apple
Source: Hortic Res. 2025 Apr 29;12(8):uhaf120. doi: 10.1093/hr/uhaf120 (PMC12261083; doi:10.1093/hr/uhaf120)
Supplement: Web_Material_uhaf120 [file web_material_uhaf120.zip › Supplemental Information-20250408.docx]

**Horticulture Research Supplemental Information for Figures 1-14**

**Two DNA-binding One Zinc Finger (DOF) transcription factors MdCDOF3 and MdDOF3.6 accelerate leaf senescence by activating cytokinin oxidase MdCKX7 in response to sorbitol signaling in apple**

Wang-Jiang Zhang^a^, Chang-Ning Ma^a^, Lian-Da Du^a^, Ying Xiang^a^, Fan Xiao^a^, Ya-Ting Liu^a^, Chu-Kun Wang^a^, Wan-Kun Li^a^, Ting-Ting Zhao^a,1^, Da-Gang Hu^a,1^

**Author Affiliations:**

^a^National Research Center for Apple Engineering and Technology; Shandong Collaborative Innovation Center of Fruit & Vegetable Quality and Efficient Production; College of Horticulture Science and Engineering, Shandong Agricultural University, Tai’an, Shandong 271018, China

**^1^Corresponding authors:**

Da-Gang Hu (hudagang@sdau.edu.cn or fap_296566@163.com), Ting-Ting Zhao [(zhaoting@sdau.edu.cn](mailto:(zhaoting@sdau.edu.cn)datingsdau@126.com)

Tel: +86-538-824-6151

Fax number: +86-538-824-6151

Address: No.61 Daizong street, Tai’an, Shandong 271018, China

**Short Title:**

Sorbitol accelerates leaf senescence via cytokinin pathway

**One-sentence Summary:**

Sorbitol orchestrates the expression of *cytokinin oxidase* within the cytokinin signaling pathway by regulating the expression profiles of two DNA-binding One Zinc Finger (DOF) transcription factors, thereby influencing cytokinin accumulation in apple leaves and ultimately accelerating leaf senescence.


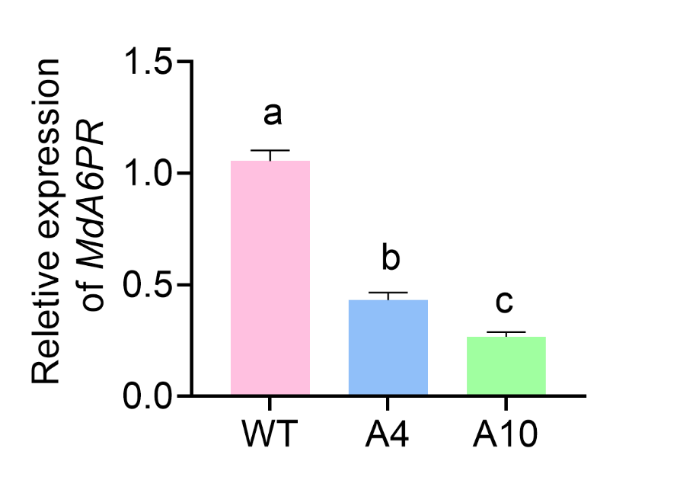


**Supplemental Figure 1. RT-qPCR assay was performed to detect the expression of *A6PR* in the wild type and two antisense lines (A4, A10).**

Data are expressed as the mean ± SEM of three biological replicates. Different letters (a and b) indicate significant differences (P < 0.05) between genotypes using Duncan’s multiple range test (MRT) after ANOVA.


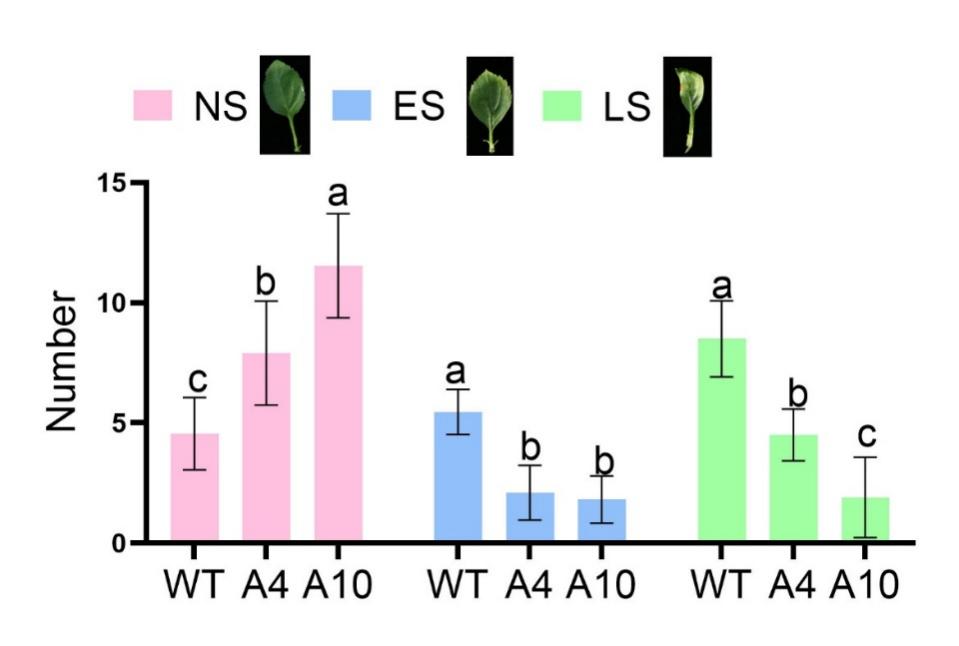


**Supplemental Figure 2. The statistical number of leaves of wild type and two antisense lines (A4, A10) in different senescence states was recorded during 40 days of succeeding culture.**

Different letters (a and b) indicate significant differences (P < 0.05) between genotypes using Duncan’s multiple range test (MRT) after ANOVA.


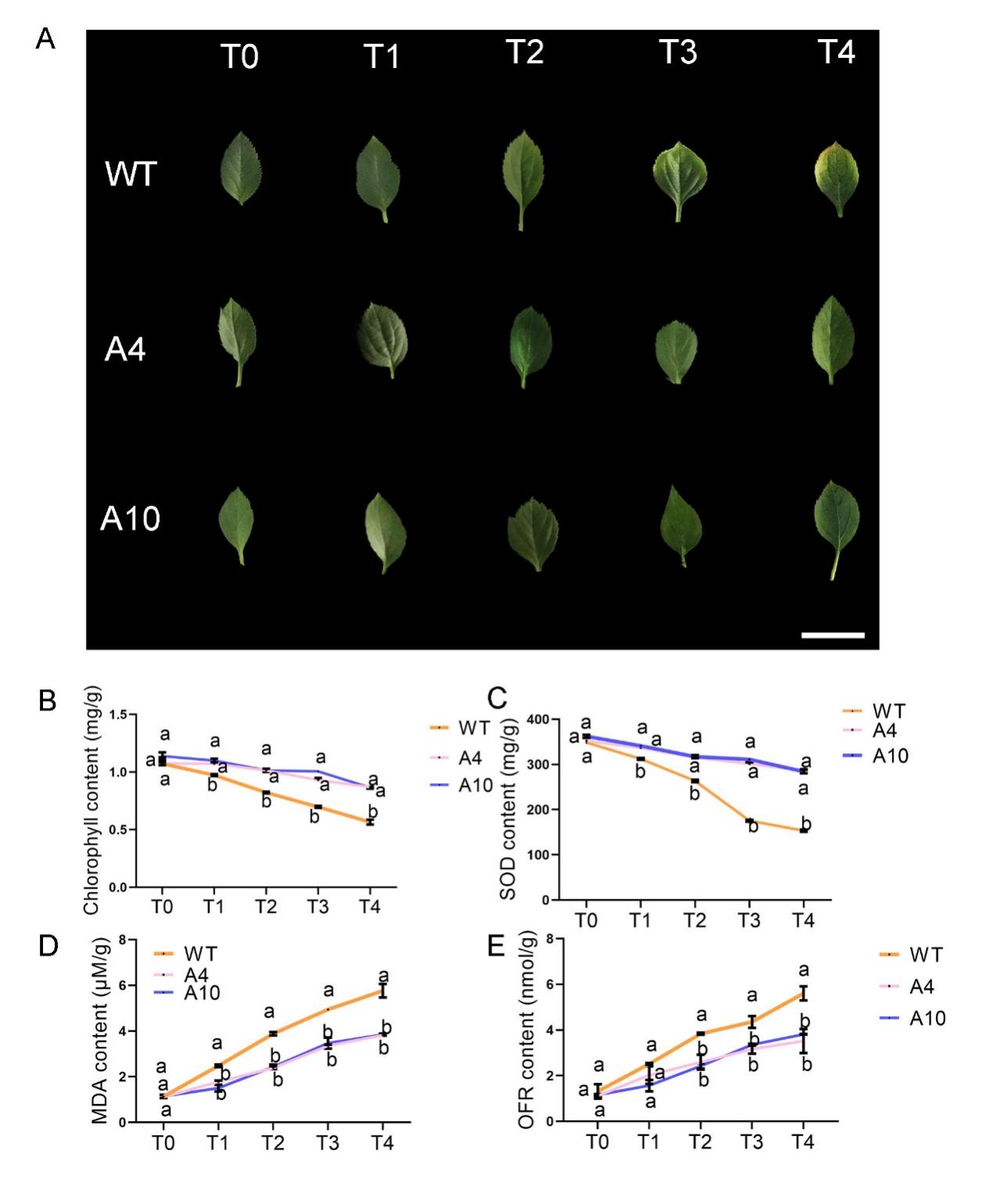


**Supplemental Figure 3. Within 50 days of succession culture, leaf alterations in chlorophyll content, superoxide dismutase (SOD) content, and malondialdehyde (MDA) levels were observed in the wild type and two antisense lines (A4 and A10) during the relay culture period.**

1. The second leaf at the base of the plant ten days after the initial succession was used for the first sampling (T0 = 10 days), and so on every ten days. Scale bar = 1 cm. (B) Chlorophyll content (mg/g FW) of WT, A4 and A10 leaves. (C) SOD content (U/g FW) of WT, A4 and A10 leaves. (D) MDA content (μM/g FW) of WT, A4 and A10 leaves. (E) OFR content (nmol/g FW) WT, A4 and A10 leaves. Data are expressed as the mean ± SEM of three biological replicates. Different letters (a and b) indicate significant differences (P < 0.05) between genotypes using Duncan’s multiple range test (MRT) after ANOVA. FW, fresh weight.


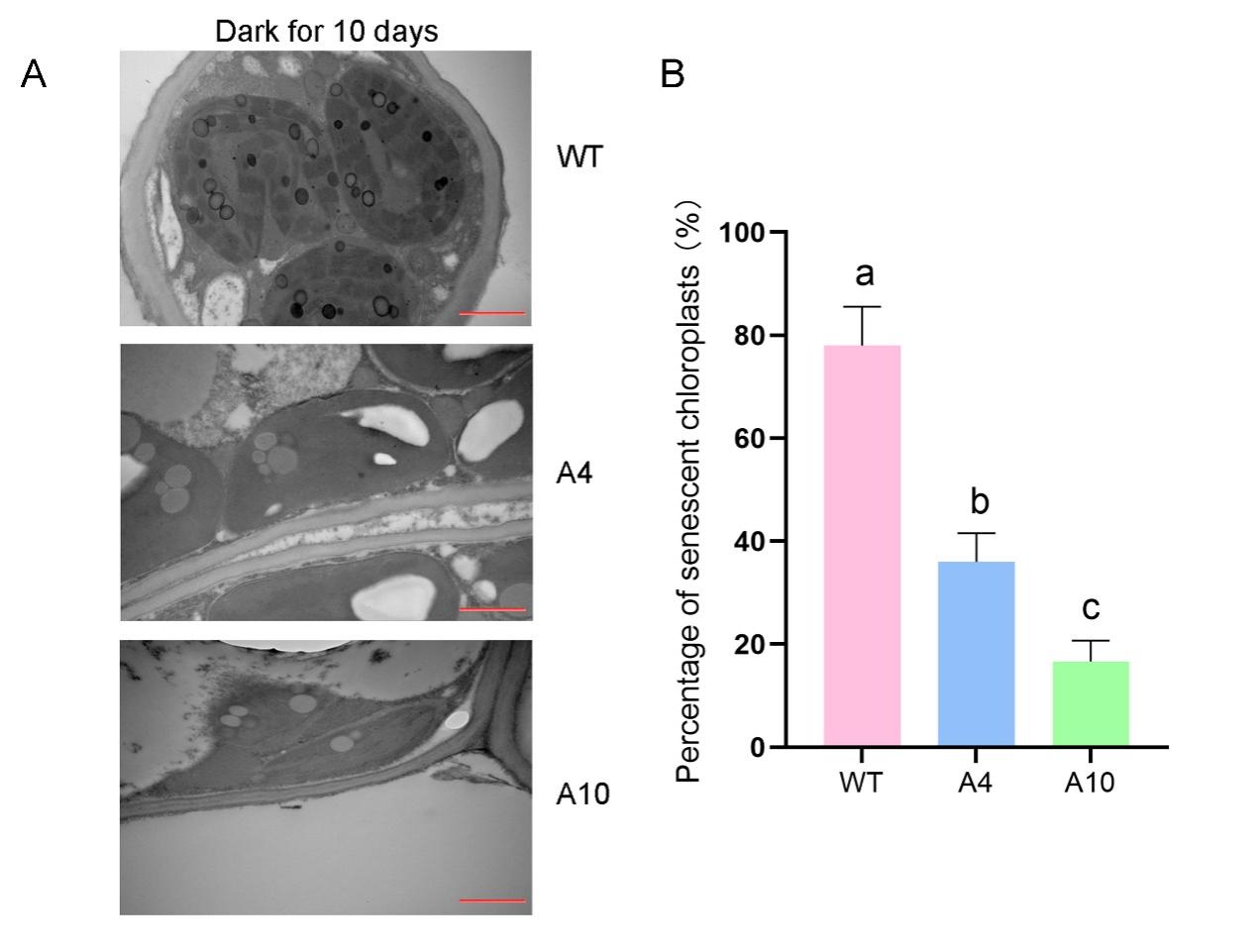
**Supplemental Figure 4. Transmission electron microscopy analysis of chloroplast morphology in WT and two antisense lines (A4 and A10) following 10-day dark treatment.**

(A) The chloroplast morphology of 10 day dark treated wild-type and A4, A10 leaves was photographed by transmission electron microscopy (TEM) observation, Scale bars = 2 μm.（B）The percentage of senescent chloroplasts to total chloroplasts in the field of view was counted at a magnification of 1500. Data are expressed as the mean ± SEM of three biological replicates. Different letters (a and b) indicate significant differences (P < 0.05) between genotypes using Duncan’s multiple range test (MRT) after ANOVA.


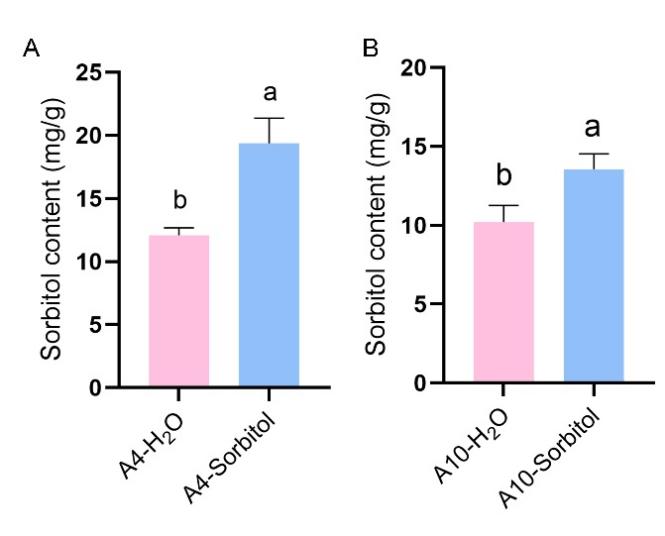


**Supplemental Figure 5. Detection of sorbitol content in A4 and A10 leaves under 50 mM sorbitol treatment.**

(A) Sorbitol content (mg/g FW) in A4 leaves treated with sorbitol solution or water (control). Data are expressed as the mean ± SEM of three biological replicates. Different letters (a and b) indicate significant differences (P < 0.05) using Duncan’s multiple range test (MRT) after ANOVA. (B) Sorbitol content (mg/g FW) in A10 leaves treated with either sorbitol solution or water (control). Data are expressed as the mean ± SEM of three biological replicates. Different letters (a and b) indicate significant differences (P < 0.05) using Duncan’s multiple range test (MRT) after ANOVA.


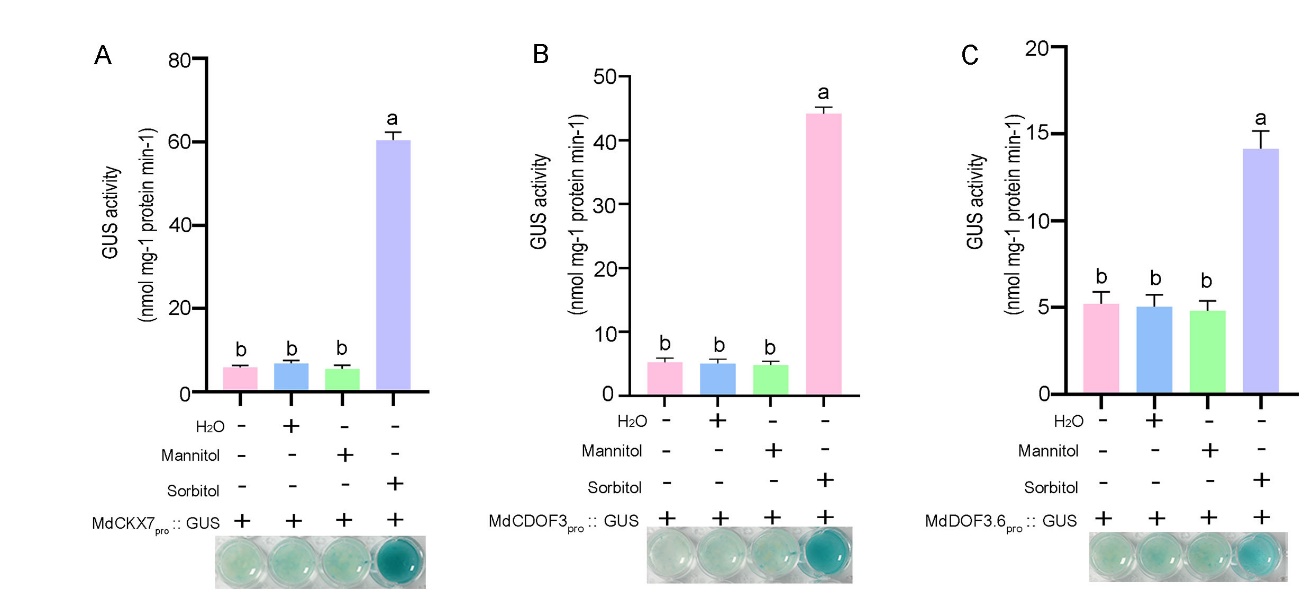


**Supplemental Figure 6.** **Apple calli GUS assay showed that sorbitol induced the expression of *MdCKX7*, *MdCDOF3*, and *MdDOF3.6.***

The 'Orin' calli were treated with 50 mM sorbitol, with H_2_O and 50 mM mannitol serving as control. Data are expressed as the mean ± SEM of three biological replicates. Different letters (a and b) indicate significant differences (P < 0.05) using Duncan’s multiple range test (MRT) after ANOVA.


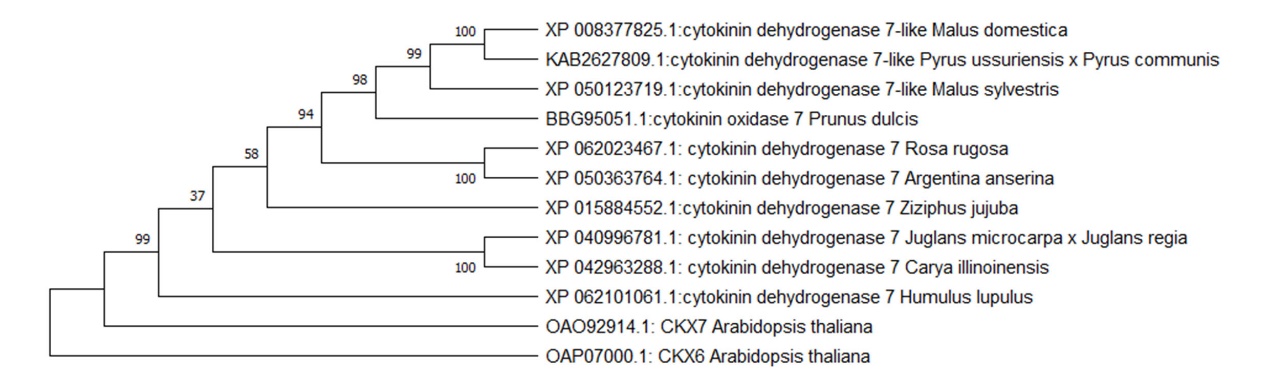


**Supplemental Figure 7. Phylogenetic tree analysis of cytokinin oxidase 7.**

The evolutionary relationships of CKX7s in various plant species were analyzed using the MEGA11 software with the maximum likelihood (ML) method. The protein sequences of CKX7s were obtained from the [National Center for Biotechnology Information](https://www.ncbi.nlm.nih.gov/) (NCBI) database and MUSCLE was used to ensure high-quality multiple sequence alignments. Bootstrap values at the nodes are percentages of 1,000 replicates. Branch lengths indicate the number of substitutions per site.


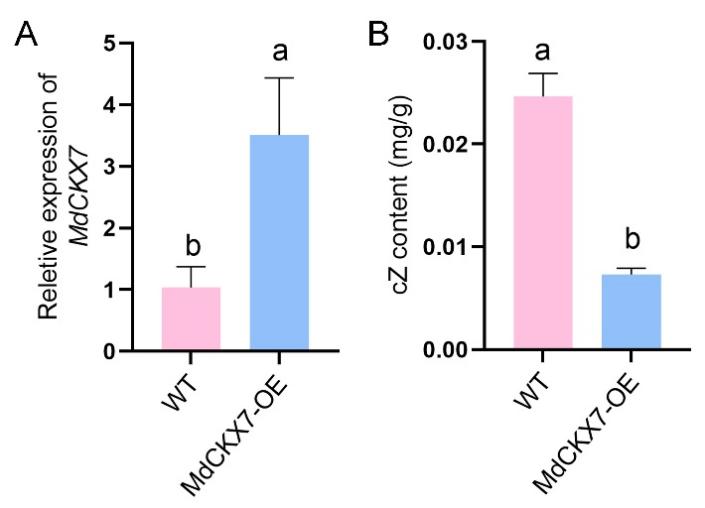


**Supplemental Figure 8. RT-qPCR validation and cZ content assay were conducted to confirm the overexpression of *MdCKX7* in transgenic apple calli.**

1. The expression level of *MdCKX7* based on RT-qPCR in *MdCKX7* overexpressing calli. (B) cZ content (mg/g FW) of the *MdCKX7* overexpressing calli. Data are expressed as the mean ± SEM of three biological replicates. Different letters (a and b) indicate significant differences (P < 0.05) between genotypes using Duncan’s multiple range test (MRT) after ANOVA.


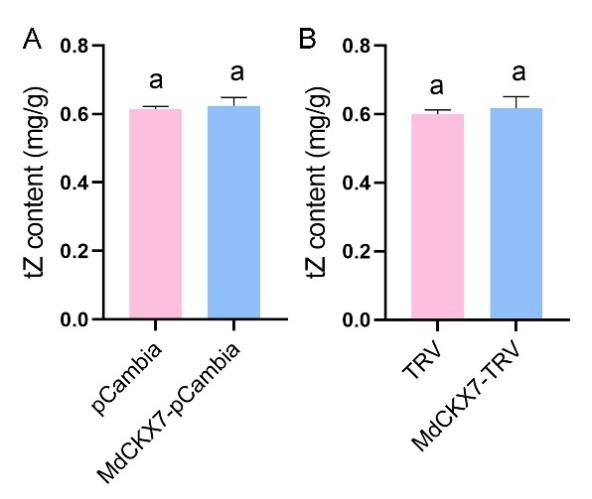


**Supplemental Figure 9.** **Detection of trans-zeatin (tZ) content in apple leaves overexpressing and silencing *MdCKX7*.**

(A) The tZ content of apple leaves overexpressing *MdCKX7* after a dark treatment for seven days, with pCambia as control. (B) The tZ content of apple leaves silencing *MdCKX7* after a dark treatment for ten days, with TRV as a control. Data are expressed as the mean ± SEM of three biological replicates. Different letters (a and b) indicate significant differences (P < 0.05) using Duncan’s multiple range test (MRT) after ANOVA.


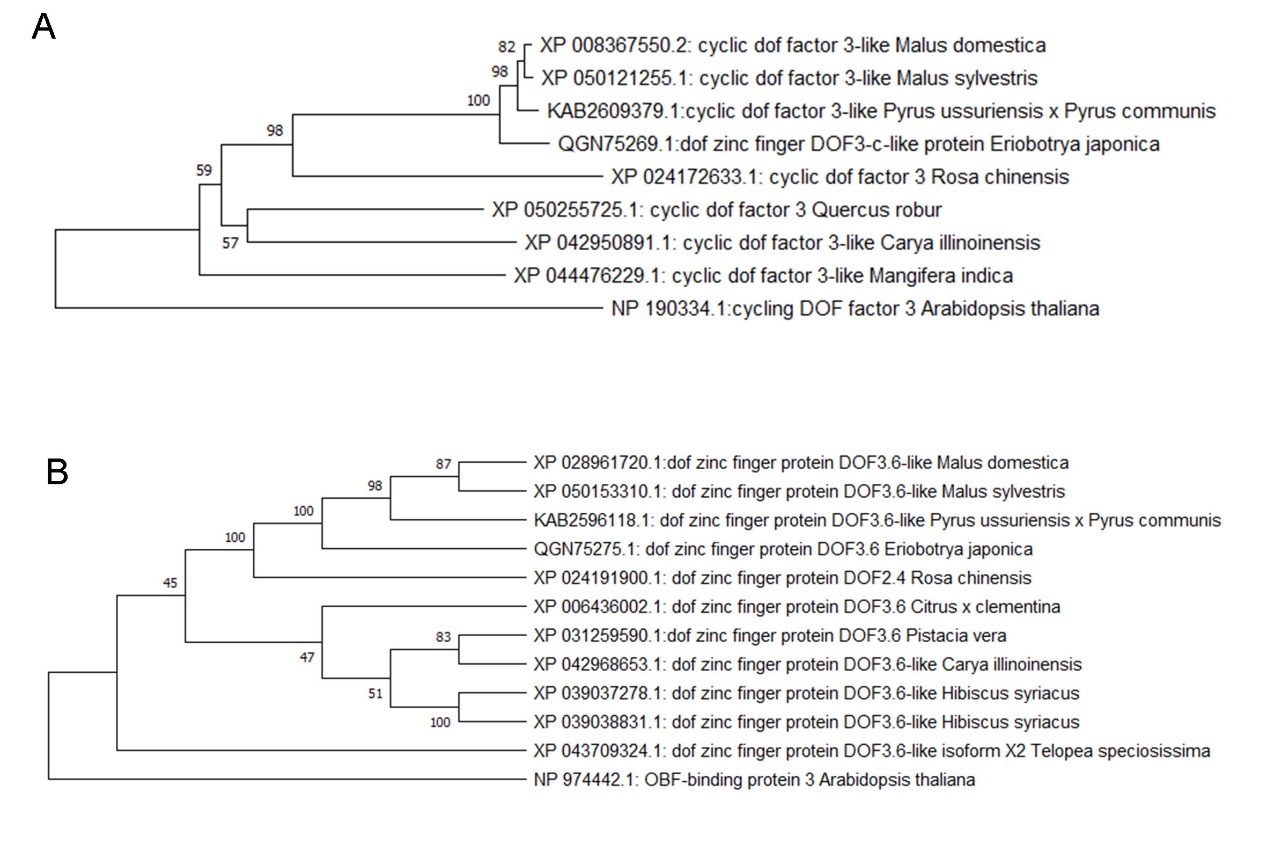


**Supplemental Figure 10. Phylogenetic tree analysis of CDOF3 and DOF3.6.**

The evolutionary relationships of CDOF3s (A) and DOF3.6s (B) in various plant species were analyzed using the MEGA11 software with the maximum likelihood (ML) method. The protein sequences of CDOFs (A) and DOF3.6s (B) were obtained from the NCBI database and MUSCLE was used to ensure high-quality multiple sequence alignments. Bootstrap values at the nodes are percentages of 1,000 replicates. Branch lengths indicate the number of substitutions per site.


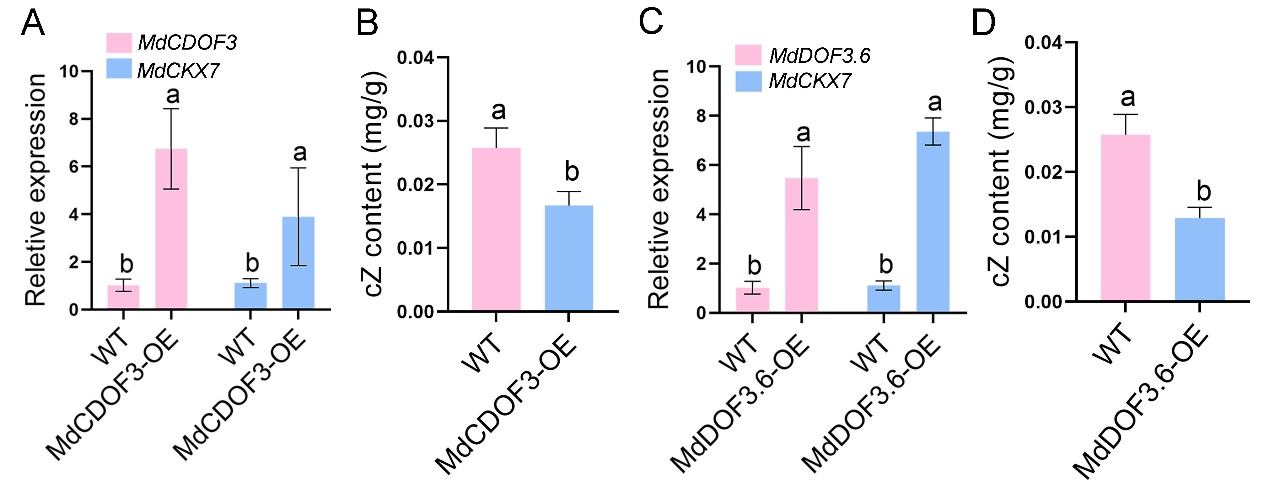


**Supplemental Figure 11. RT-qPCR was performed to identify the overexpression of *MdCDOF3* and *MdDOF3.6* in apple calli, and the cZ content was also detected.**

(A) The expression levels of *MdCDOF3* and *MdCKX7* based on RT-qPCR in *MdCDOF3* overexpressing calli. (B) The content of cZ (mg/g FW) in *MdCDOF3* overexpressing calli. (C) The expression levels of *MdDOF3.6* and *MdCKX7* based on RT-qPCR in *MdDOF3.6* overexpressing calli. (D) The content of cZ content (mg/g FW) in *MdDOF3.6* overexpressing calli. Data are expressed as the mean ± SEM of three biological replicates. Different letters (a and b) indicate significant differences (P < 0.05) between genotypes using Duncan’s multiple range test (MRT) after ANOVA.


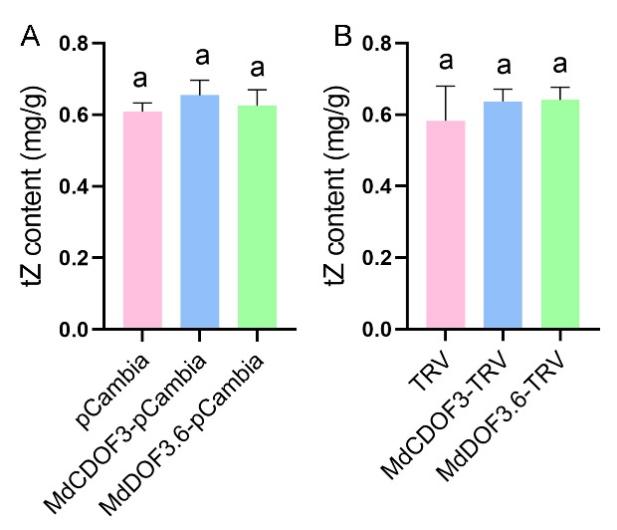


**Supplemental Figure 12.** **Detection of trans-zeatin (tZ) content in apple leaves overexpressing and silencing *MdCDOF3* and *MdDOF3.6.***

(A) The tZ content of apple leaves overexpressing *MdCDOF3* and *MdDOF3.6* after a dark treatment for seven days, with pCambia as control. (B) The tZ content of apple leaves silencing *MdCDOF3* and *MdDOF3.6* after a dark treatment for ten days, with TRV as a control. Data are expressed as the mean ± SEM of three biological replicates. Different letters (a, b and c) indicate significant differences (P < 0.05) using Duncan’s multiple range test (MRT) after ANOVA.

**
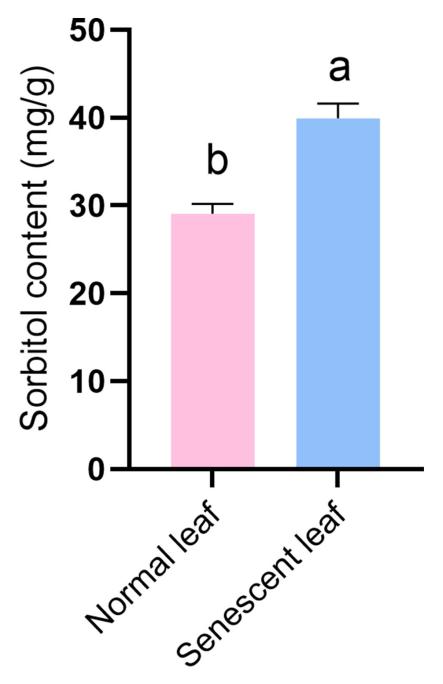
**

**Supplemental Figure 13. The sorbitol content in normal and senescent leaves.**

Sorbitol content (mg/g FW) of the normal leaf and senescent leaf. Data are expressed as the mean ± SEM of three biological replicates. Different letters (a and b) indicate significant differences (P < 0.05) between genotypes using Duncan’s multiple range test (MRT) after ANOVA.


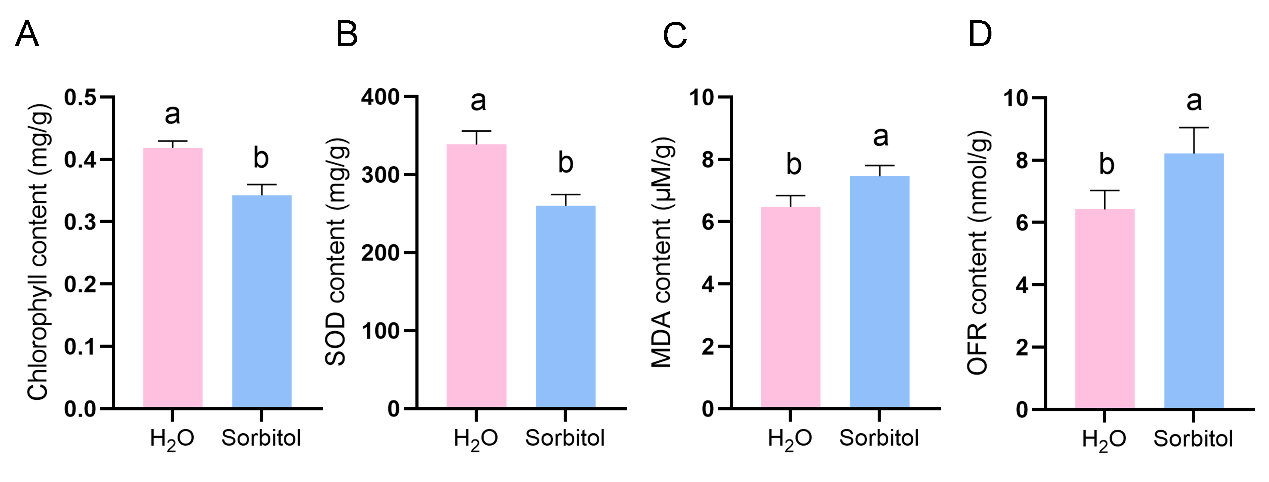


**Supplemental Figure 14.** **Chlorophyll, superoxide dismutase (SOD), malondialdehyde (MDA) , and oxidative free radical (OFR) contents of wild-type leaves fed with 50mM sorbitol.**

1. D) Chlorophyll content (A), SOD content (B), MDA content (C), and OFR content (D) of apple leaves fed with 50mM sorbitol and H_2_O. Data are expressed as the mean ± SEM of three biological replicates. Different letters (a and b) indicate significant differences (P < 0.05) between genotypes using Duncan’s multiple range test (MRT) after ANOVA. FW, fresh weight.
